# Health system facilitators and barriers to the integration of mental health services into primary care in the Democratic Republic of the Congo: a multimethod study

**DOI:** 10.1186/s12875-024-02460-y

**Published:** 2024-06-13

**Authors:** Erick Mukala Mayoyo, Faustin Chenge, Abdoulaye Sow, Bart Criel, Joris Michielsen, Kris Van den Broeck, Yves Coppieters

**Affiliations:** 1 School of Public Health, University of Lubumbashi, Lubumbashi, Democratic Republic of the Congo; 2 Research Center in Epidemiology, Biostatistics and Clinical Research, School of Public Health, Université Libre de Bruxelles, Brussels, Belgium; 3 Department of Community Health, Institut Supérieur des Techniques Médicales de Kananga, Kananga, Democratic Republic of the Congo; 4 Centre de Connaissances en Santé en RD Congo, Kinshasa, Democratic Republic of the Congo; 5 Faculty of Health Science and Techniques, Gamal Abdel Nasser University of Conakry, Conakry, Guinea; 6 Department of Public Health, Institute of Tropical Medicine Antwerp, Antwerp, Belgium; 7 Department of Family Medicine and Population Health, Faculty of Medicine and Health Sciences, University of Antwerp, Antwerp, Belgium

**Keywords:** Mental health services, Primary care, Integration, Health system, Facilitator, Barrier, Multimethod study, Democratic Republic of the Congo

## Abstract

**Background:**

The integration of mental health into primary care—i.e., the process by which a range of essential mental health care and services are made available in existing multipurpose health care settings that did not previously provide them—can be facilitated or hindered by several health system factors that are still poorly understood. This study aimed to identify health system facilitators and barriers to the integration of mental health services into primary care in the Democratic Republic of the Congo (DRC) to improve the success rate of integration programs.

**Methods:**

We conducted a multimethod, cross-sectional exploratory study. Stakeholders (managers, health service providers, service users, etc.) from sixteen of the twenty-six provinces of the DRC participated. We collected qualitative data through 31 individual, semistructured, face-to-face key informant interviews. We then collected quantitative data through a population-based survey of 413 respondents. We analyzed the interviews via thematic analysis, assigning verbatims to predefined themes and subthemes. For the survey responses, we performed descriptive analysis followed by binomial logistic regression to explore the associations between the variables of interest.

**Results:**

Strong leadership commitment, positive attitudes toward mental health care, the availability of care protocols, mental health task sharing (*p* < 0.001), and sufficient numbers of primary care providers (PCPs) (*p* < 0.001) were identified as key health system facilitators of successful integration. However, barriers to integration are mainly related to a poor understanding of what integration is and what it is not, as well as to the poor functionality and performance of health facilities. In addition, stigma, low prioritization of mental health, lack of mental health referents, low retention rate of trained health professionals, lack of reporting tools, lack of standardized national guidelines for integration (*p* < 0.001), lack of funding (*p* < 0.001), shortage of mental health specialists to coach PCPs (*p* < 0.001), and lack of psychotropic medications (*p* < 0.001) were identified as health system barriers to integration.

**Conclusion:**

Improving the functionality of primary care settings before integrating mental health care would be beneficial for greater success. In addition, addressing identified barriers, such as lack of funding and mental health-related stigma, requires multistakeholder action across all building blocks of the health system.

**Supplementary Information:**

The online version contains supplementary material available at 10.1186/s12875-024-02460-y.

## Background

Mental health disorders are highly prevalent, particularly in sub-Saharan Africa (SSA) and the Democratic Republic of the Congo (DRC). In 2019, the SSA region accounted for 14% of the global burden of mental disorders, with the disability-adjusted life years (DALYs) rate (per 100,000 people) estimated at 1,397.31 [1]. In addition, the prevalence of mental disorders in the DRC was estimated at 12.09%, the DALY rate (per 100,000 population) was estimated at 1,577.7, and the age-standardized suicide mortality rate (per 100,000 population) was estimated at 12.41; health expenditures were mainly development aid (46.4%) and out-of-pocket expenditures (35.4%), and the effective coverage rate of essential services provision for universal health coverage (UHC) was 45.2% [1–3]. The United Nations (UN) has good intentions to conquer these challenges, e.g., the UN Sustainable Development Goals (SDGs), including SDG3 (Ensure healthy lives and promote well-being for all at all ages), especially targets 3.4, 3.5, and 3.8 on mental health and well-being, substance abuse, and universal health coverage (UHC), respectively. To achieve progress, it is necessary to ensure that all individuals and communities have access to quality, safe, and acceptable (mental) health services [4, 5]. In a context where the coverage of mental health services in primary care is less than 10%, integrating mental health services into primary care could fill treatment gaps [6–8].

In this paper, we defined mental health integration as the process of incorporating—according to a set of context-appropriate models and techniques—a basic package of curative, preventive, promotional, and rehabilitative mental health and psychosocial support services into the primary care system, specifically the complementary package of activities offered in a district hospital (i.e., the primary referral health facility) and the minimum package of activities offered in health centers (i.e., frontline health facilities), as well as community-based activities offered at the community level (i.e., a zero-level system) [9]. Its aim is not to incorporate an entire vertical program into existing general health services but rather to integrate targeted health and social care interventions or activities, or even program tasks, into the activity packages of health facilities offering primary care [9–12]. The integration process involves building the capacity of primary care providers (PCPs), shaping their attitudes, and shifting certain mental health-related tasks to them. This process may be supported by health system facilitators or hindered by health system barriers, which remain poorly understood in the context of the DRC. Drawing on research by Auschra [13], we defined a health system barrier as any health system-related factor that may hinder the integration of mental health into primary care services. In contrast, a health system facilitator is any health system-related factor that may facilitate the integration of mental health into primary care services.

In most low- and middle-income countries (LMICs), achieving UN SDG3 targets 3.4, 3.5, and 3.8 through the integration of services based on well-developed social security and social protection systems can be challenging. For instance, in the DRC, human capacity is lacking, and financial means are not optimally used in the domain of health. For the mental health sector, this resulted in only six recognized psychiatric hospitals, with a capacity of 500 hospital beds, i.e., 1 bed per 190,000 inhabitants, and a few private mental health centers [14]. In practice, due to the lack of formal mental health provisions in the primary healthcare system, traditional medicine and spiritual healing have, until recently, been the main sources of care for mental health problems [15]. To fill this treatment gap, the first well-documented program to effectively integrate mental health into primary care was officially launched in 2011 in the health district of Lubero, North Kivu Province [16], a rural area characterized by recurrent armed conflicts.

Nevertheless, the DRC has an ambitious national mental health plan, and the Ministry of Health has decided to invest in mental health [17] to fill the treatment gaps in this area. There are several ongoing programs in this regard. An integration program supported by the Institute of Tropical Medicine (ITM) in Antwerp through its 4th Framework Agreement funded by the Directorate General for Development Cooperation (DGD) was launched in May 2021 in the Tshamilemba district, Lubumbashi, Haut-Katanga Province. Another, financed by Memisa (a Belgian, not-for-profit, nongovernmental organization), was launched in December 2022 in the rural district of Mangembo in Kongo Central Province. These integration programs have been implemented to provide primary mental health care to the population using the health systems strengthening (HSS) approach [18]. They aim to shift mental health care from a psychiatric hospital-based model—once used in the DRC—to an outpatient primary health and social care model with improved quality and community-based care and to build a continuum of mental health care from the community to health centers and district hospitals. Since 2011, the National Mental Health Program (PNSM) has promoted the integration of mental health, in line with the mental health Gap Action Program (mhGAP) policy launched by the World Health Organization (WHO) in 2008 [16].

To maximize the success rate of ongoing and future integration programs, it is important to understand the health system facilitators and barriers that may help/hinder such a transition from a hospital-based model to an ambulatory primary care service. A number of internal and external health systems and contextual factors have been identified in the literature [19–21]. Internal factors include the degree of preparation, planning, and implementation of the integration process; the precise nature of the complex set of user needs; the knowledge and skills of providers; the motivation for change; the nature of health care management; the availability of medications; the responsiveness of health care structures to the needs of the population; the effectiveness of primary care facilities in managing mental disorders; the availability of PCPs; and the presence of mental health specialists. Exogenous factors include changes in the number of health facilities and population density in the health district. Finally, contextual factors include mental health-related stigma; the perceptions and attitudes of various stakeholders regarding the acceptability, relevance, and credibility of the integration program; the budget allocated to mental health; the purchasing power of users; and various beliefs regarding the origin of mental illness. When well-conceived and implemented, integration can strengthen the (mental) health system [22, 23], i.e., it considers the influence of both endogenous and exogenous factors on the system. However, less attention has been given to exogenous factors in the healthcare system [24], particularly those focusing on the integration of mental health into primary care in our context.

The DRC, which serves as the context for the integration programs analyzed, is a low-income SSA country with a gross national income per capita of less than $1,139 [25]. The Congolese health system, which in 2021 covered the health needs of approximately 95,000,000 inhabitants, is subdivided into 519 health districts, with at least 133,373 health facilities, the majority of which have activity packages that exclude mental health provisions [26].

However, since 1999, the country has had an ambitious mental health plan, followed by the creation of the PNSM (AM: 1250/CAB/MIN/S/AJ/008/2001) [18]. The PNSM has not yet significantly fulfilled its mission of promoting mental health throughout the country due to a lack of sufficient human and financial resources. For example, there are currently 0.10 psychiatrists, 0.25 mental health nurses and 0.02 psychologists per 100,000 people [27]. In addition, apart from the government, which pays the salaries of civil servants, the PNSM receives occasional technical and financial support from its partners recognized by program managers. These partners include UN agencies (e.g., WHO), development cooperation agencies (e.g., Enabel), international and local NGOs (e.g., Fracarita, Open Society Initiative for Southern Africa, Christian Blind Mission, Memisa, Panzi Foundation, etc.), and academic institutions (ITM, etc.).

To extend the coverage of mental health services, the PNSM management team revised the subsector national mental health policy in 2021. One of the strategic axes of this policy is the integration of mental health into primary care settings and continuity of care [18]. These district health services, notably the health center, constitute the gateway to the health system and, in some provinces, are used by 43–79% of patients as first or second resorts [28]. As the Ministry of Health and its partners work to improve the population coverage of mental health services by integrating mental health into primary care, there is a need to better understand the health system factors that may facilitate or hinder the success of integration programs. This study aimed to identify health system factors that may facilitate or hinder the integration of mental health into the Congolese primary care system to fill the evidence gap on this topic.

## Materials and methods

### Study design and setting

We conducted a multimethod, cross-sectional exploratory study. The exploratory qualitative part, using content analysis, explored stakeholders’ perceptions of health system factors that may facilitate or hinder the integration of mental health care into primary care services. The quantitative part, using a cross-sectional design, examined the associations between these health system factors and the feasibility (or not) of this integration.

Surveys and interviews were conducted in the country’s two largest cities, Kinshasa and Lubumbashi. Kinshasa is the administrative capital of the country, with approximately 16,316,000 inhabitants in 2022 [29], while Lubumbashi is the economic capital, with approximately 2,695,000 inhabitants during the same year [30]. The Kinshasa site was chosen because it hosts a high-level national advocacy workshop for integrating mental health into primary health care (PHC) in the DRC. The PNSM held this workshop from December 10 to 12, 2022. Over one hundred participants, including decision makers, health managers, and health system development partners, from 16 of the country’s 26 provinces, including those from Haut-Katanga Province, participated. In Kinshasa city, the study population comprised participants in this national workshop. We took advantage of the opportunity provided by the presence of workshop delegates to collect data from key informants (KIs) on the topic. Stakeholders from 10 of the remaining 26 provinces in the country who had no experience with integration or no plans for integration in the short or medium term were not invited to participate in the national workshop and were therefore not included. However, the Lubumbashi site was deliberately selected because there is, in this city, an ongoing mental health integration program that will be launched in 2021, as mentioned above.

### Conceptual framework

**Fig. 1:**
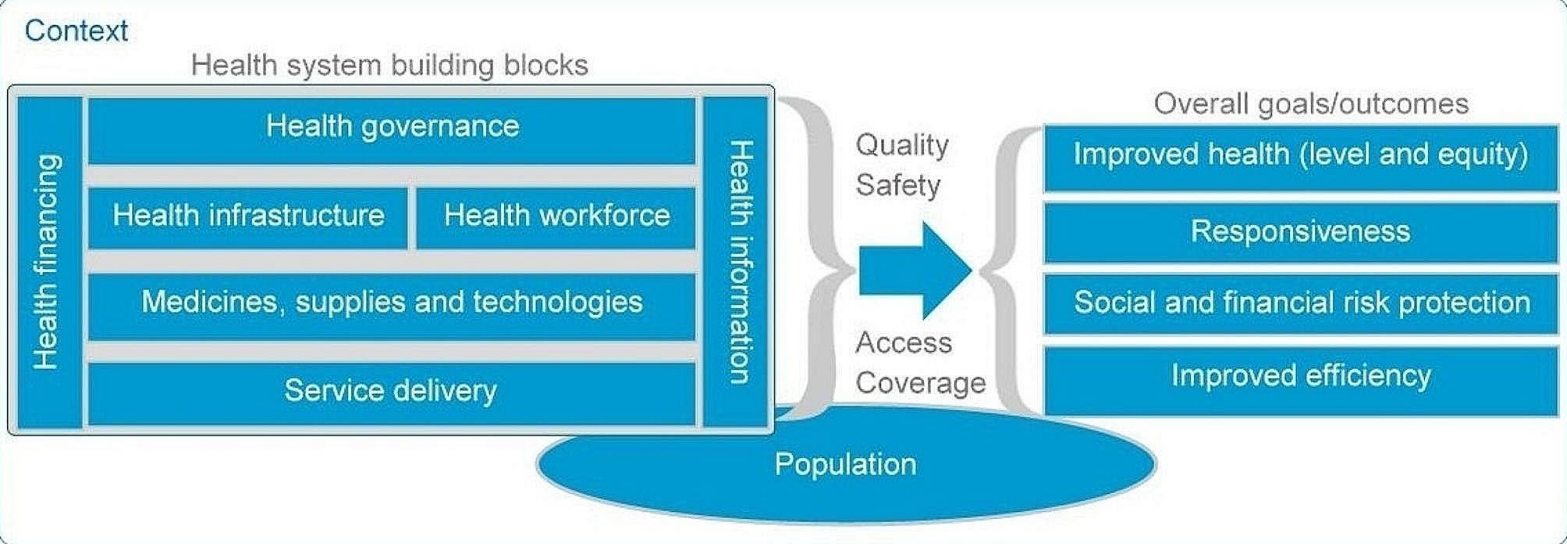
The WHO building blocks framework. Adapted from the WHO [23] and the WHO Regional Committee for Africa [31]

In this study, we adopted the WHO building blocks framework [23]. It was chosen to guide the analysis, specifically to organize and code the qualitative data and to drive the thematic analysis and structuring of the findings. The revised version of this WHO framework describes the health system in terms of seven building blocks—governance, human resources, financing, medicines and technologies, service delivery, information, and infrastructure [23, 31]—that respond to the needs of the population [32] in a given context (Fig. 1).

Health system building blocks are interdependent. As a result, interventions in some building blocks will have (un)intended consequences on other blocks that depend on them [24]. For example, to put into practice the knowledge of integration acquired during on-the-job training, providers must be well motivated, drugs must be available, the population must be informed about the existence of health services, governance must be improved, and the health services/system must be adequately financed [33]. This WHO framework conveys ideas of complexity and systems thinking in health commonly used to study complex social interventions that are conducted in health systems aimed at HSS [33], such as the implementation of mental health integration.

### Population and sampling

The study population from which the qualitative and quantitative samples described below were drawn included several key stakeholders in the integration of services at the national, provincial, and local levels of the health system. The participants, from sixteen provinces, were gathered in either Kinshasa or Lubumbashi and were divided into two distinct subpopulations. The subpopulation in Kinshasa included residents of the capital city and provinces, both urban and rural, who participated in the national workshop. The respondents who participated in the qualitative interviews and the quantitative survey in Kinshasa no longer participated in those in Lubumbashi. However, in both cities, the selected respondents participated in both qualitative and quantitative surveys.

In Lubumbashi, the subpopulation consisted of a range of health system stakeholders, including decision makers, health managers, service providers, service users (e.g., people living experience of mental health conditions, family heads), and health system development partners, all of whom lived in the city. These stakeholders were identified through district managers and community health workers (CHWs). In Kinshasa, the subpopulation comprised key informants (i.e., decision makers, health managers, health system development partners) from the national workshop mentioned above, service providers, and service users.

To participate in the surveys, participants had to meet the following criteria: (i) be 18 + years old, (ii) be able to read and write in French, Kiswahili, and/or Lingala, and (iii) freely consent to participate. Those who had not reached the age of majority by the day of the surveys and who were not directly involved in the integration of mental health despite being in the system were excluded.

We constructed the following two samples: an opportunistic and purposive sample [20] composed of stakeholders/KIs for the qualitative approach and a simple random sample for the quantitative approach.

#### Qualitative sample

This was an opportunistic and purposive sample where we met KIs who had attended the national workshop in Kinshasa as provincial delegates from their institutions/organizations. A total of 44 invited KIs agreed to respond, but data saturation [20] was reached at 31 interviews, requiring data collection to be stopped and the sample size to be determined. The socioprofessional profile of these 31 KIs is described in the results section below.

#### Quantitative sample

We used a simple random sample of participants to account for the diversity of health system stakeholders (i.e., policy makers, health managers, service providers, service users, and health system development partners) and to provide statistical power at the subgroup level. To estimate the optimal number of participants, the following formula [34] was used: n = Za^2^*(p*q)/(d^2^), where Z*a*^2^ (i.e., confidence interval coefficient, squared) = 1.96², *p* = 0.5 and q = 0.5 (as the proportion of people reporting that mental health is integrated into primary care is not known in the context of the study, 50% are considered to be in favor and 50% are not), and d^2^ (statistical precision) = 0.05². After calculation, the minimum sample size was set at 385 subjects. To account for the nonresponse fraction, this sample size was increased by 10% because this was not a social desirability study, resulting in a sample size of 424 subjects. However, out of all participants expected, the response rate was 98.1%.

Once they were identified as potential participants, we invited them to participate in the study. The CHWs spread the invitation orally by word of mouth. Most of them accepted the invitation as described above.

### Methods and data collection

The first part of the data collection was conducted in Kinshasa from December 10 to 13, 2022, and the second part was conducted in Lubumbashi from July to November 2023. We used the WHO building blocks framework to develop the data collection tools and organize the interview content.

#### Methods and qualitative data

We conducted individual, semistructured, face-to-face key informant interviews (KIIs). The interview method is recommended for exploring research questions for which there is little or no prior evidence [35]; in this case, there is a lack of evidence on health system facilitators and barriers to the integration of mental health into primary care in the DRC context. The interview guide, appended as a supplementary file (Text S1), was developed in French. This approach was used to organize our semistructured interviews with KIs. This tool was pretested in Lubumbashi, with 6 KIs not included in the study. This pretest helped us reformulate some questions to make them clearer by including prompts relating to each of the health system building blocks.

For urban transport reasons, we conducted KIIs from Monday to Saturday, from 8 a.m. to 5 p.m. At the participants’ request, the meeting places were either work offices, homes, or other locations at their convenience. The KIIs, which lasted an average of 60 min, were recorded using a dictaphone with the participants’ consent. The principal investigator conducted the interviews in both cities, while the research assistant was responsible for audio-recording the interviews and taking field notes. The interview guide was iteratively adapted throughout the process according to the relevance of the information provided.

The recorded data were transcribed verbatim by the research assistants and checked by the principal investigator against the audio recordings made to ensure accuracy before moving on to the next series of interviews. The transcripts were then entered and stored in an NVivo software database, which enabled qualitative analysis. The quality and accuracy of the transcripts were checked by listening to all recordings and correcting them where necessary.

#### Methods and quantitative data

We used a population-based survey supported by a Likert-type questionnaire (with 5 items ranging from 1. strongly disagree to 5. strongly agree). This individual survey questionnaire, appended as a supplementary file (Text S2), listed several factors likely to facilitate or hinder the implementation of health service integration, including mental health services, and grouped them according to health system building blocks. It also included information on socioprofessional characteristics and opinions on mental health integration. Before the pretest, the questionnaire, which was prepared in its preliminary version in French, was translated into local languages (Kiswahili and Lingala) and then back-translated into French by a sworn translator. Thus, the questionnaire was pretested in Lubumbashi with 18 stakeholders not included in the study. This ensured the acceptability and comprehensibility of the questions. No major changes were made following this pretest.

Before the survey, eight locally recruited interviewers, four in each of the two cities (Kinshasa and Lubumbashi), including two women and all health professionals with at least three years of experience in the Congolese health system, were trained. In each city, a 4-h briefing session was organized for them, led by the principal investigator, covering data collection tools and techniques.

The same questionnaire was used for both health system key informants (e.g. managers, health professionals) and service users, under the supervision of an investigator who provided clarification when needed. Completing the questionnaire took an average of 30 min. The principal investigator supervised the data collection. He compiled and coded the completed questionnaires before computer processing.

We initially decided to use a five-point Likert questionnaire to minimize the risk of acquiescence bias during data collection. Because the questionnaire items used referred to multimodal and numerical categorical variables, recoding was performed after data collection to facilitate descriptive and regression statistical analyses. For example, for the health system facilitator and barrier variables, items 1 to 3 (strongly disagree, disagree, and neither agree nor disagree) were grouped into item 1 (‘disagree’), and items 4 and 5 (‘agree, strongly agree’) were grouped into item 2 (‘agree’). These variables included dimensions related to health system building blocks and attributes to be measured. After recording, the data were exported to SPSS software for analysis.

The collected data were entered and stored in Excel by the principal investigator, where they were processed to correct outliers. During data cleaning, three observations (IDs 28, 141, and 172) were removed from the database because they contained several outliers and missing data. Therefore, after cleaning the file, the sample size was set at 413 participants, who were the subjects of our statistical analysis.

### Data analysis

#### Qualitative analysis

Drawing inspiration from the WHO framework described above, we organized and coded the data collected into two main themes: health system facilitators and health system barriers. Using NVivo software, we conducted thematic analysis and then assigned the relevant verbatims (i.e., the factors explored) extracted to each of the corresponding predefined themes and subthemes related to health system building blocks and the population. We synthesized the findings according to these themes and subthemes, specifying for whom and at what system level these factors would act as enablers and obstacles. With the support of a research assistant, the principal investigator developed codes to separate perceived facilitators and barriers related to health system building blocks. This categorical coding was performed iteratively until an agreement was reached on which responses constituted appropriate barriers or facilitators. Before coding, we consulted with two independent health system researchers to obtain their perspectives.

#### Quantitative analysis

Using SPSS software, we described the characteristics of the study participants by estimating occurrences and converting them into percentages. To estimate the probability of presumed facilitators and barriers being associated (or not) with integration, we used a binomial logistic regression with Wald’s chi-square test (Wald-χ^2^). This enabled us to identify those that were statistically significant predictors of integration success or failure. Adjusted odds ratios (aORs) were used to measure the magnitude of the associations identified. The results were deemed significant at the 5% uncertainty level (*p* < 0.05).

### Data management and ethics

This study is part of a doctoral protocol approved by the medical ethics committee of the University of Lubumbashi (UNILU/CEM/034/2021) and the Institutional Review Board of the Institute of Tropical Medicine in Antwerp (IRB/RR/AC/187/1468a/21). Before taking part in the study, all participants provided their informed and signed consent either by themselves or by delegation. During the study, we ensured compliance with ethical principles and research integrity following the Declaration of Helsinki. The interviewers administered anonymous questionnaires. Once the codes were assigned to the completed questionnaires, it was no longer possible to identify the participants. In addition, the research assistants did not have access to the files and databases after coding because they were stored on the principal investigator’s password-protected laptops. Each participant was given a unique code linked to their name on the consent form, enabling them to withdraw from the study if necessary. The authors agreed that the data would be kept for up to one year after publication of the results.

## Results

Apart from the profiles of the interviews and survey participants described below, the qualitative findings were grouped into two predefined themes—facilitators and barriers—and eight predefined subthemes—seven of which are based on health system building blocks—governance, human resources, financing, medicines, service delivery, infrastructure, and information—and the eighth added subtheme related to the population. Then, for the quantitative results, the independent variables are described, followed by those of the multivariate analysis.

### Distribution of the stakeholders interviewed

A total of 31 KIs participated in the interviews in Kinshasa. Of these KIs, 7 were managers and staff from development partner organizations, including international NGOs; 5 were managers and staff from national NGOs; 5 were provincial mental health coordinators; three were decision makers from the Ministry of Public Health; 3 were health district managers; and 3 were community leaders, household heads, and patient-service users (Table 1).

**Table 1:** Stakeholders identified and interviewed in Kinshasa and Lubumbashi (*n* = 31)

Health system level	Stakeholders	Number (Participant code, *P*#)	Seniority
**National**			
	Policymakers, Ministry of Public Health	3 (P19, P22, P24)	9–13
	Managers and staff, Development partners	7 (P16, P17, P20, P25, P26, P27, P28)	5–8
	National NGOs’ Managers and implementers	5 (P5, P8, P18, P30, P31)	4–13
	Psychiatrist, University Psychiatric Hospital	1 (P23)	14
**Provincial**			
	Coordinators, National Mental Health Program	5 (P13, P14, P15, P11, P21)	6–22
	Manager, Provincial Health Division	1 (P7)	12
**Health district**			
	Managers, Health District	3 (P2, P4, P6)	6–16
**Health area**			
	Healthcare providers, Primary care services	2 (P1, P3)	5–9
	Community leaders, household heads, and patient service users	3 (P9, P10, P12)	n. a.
**Other: University**			
	Senior Lecturer, School of Public Health	1 (P29)	7

P#, participant code; NGO, nongovernmental organization; n. a., not applicable

### Socioprofessional characteristics of the survey participants

Out of 413 subjects in the sample, 253 (61.3%) participated in Kinshasa, and 160 (38.7%) participated in Lubumbashi. The majority of respondents (65.6%) were male, 44.3% of participants were between 48 and 57 years old, and 27.1% were between 58 and 67 years old. More than half of the participants (66.3%) had a bachelor’s degree, while 24.5% had an undergraduate degree. Nearly half (48.7%) worked in the public sector, and one in three (29.5%) worked in the private, for-profit sector. In terms of primary work experience, 55.2% of the participants worked in health care, and 25.7% of the 55% working in health care were in public health. In terms of seniority, the relative majority of participants (45%) were between 10 and 14 years, while 37% were between 5 and 9. Of the respondents, 29.8% were health managers, 28.1% were healthcare providers, 18.6% were policymakers, 10.4% were health system development partners, 7.5% were implementers, and 5.6% were service users.

### Qualitative findings

When asked how stakeholders understood the integration of mental health services into primary care, we heard a variety of statements along the following lines: integrating an entire mental health system into the front line of health care; bringing people living experience of mental health conditions into the health center; making mental health accessible at the primary care level; or making care accessible throughout the primary care health system.

A few respondents understood integration in some way, as one stated:

*Integrating mental health into the first line of care means adding the mental health care management package to the existing primary care package. (P3, healthcare provider, primary care service)*

Others, however, see it differently. As one informant put it:

*Mental health integration is the addition of a service that addresses mental pathologies to the first line of care. (P6, health officer, health district)*

### Facilitators and barriers to integration by health system building block

#### Health system facilitators

##### Governance

When asked about governance-related enablers, the KI mentioned the existence of a mental health service coverage plan to guide health district coverage, as well as political will and managerial commitment. He explained it as follows:

*As coverage is very low at the national level, I think that the existence of a mental health services coverage plan drawn up by the PNSM, the will of decision-makers at all levels of the country, and the commitment of program managers are factors that can facilitate integration. (P19, policymaker, ministry of public health)*

For one KI interviewee, the availability and accessibility of national guidelines on mental health integration, task sharing, and strong leadership are factors that make it easier for program managers to follow the process in the field. Her statement reads as follows:

*Among the success factors, I would cite the availability and accessibility at all levels of clear guidelines on the integration of mental health, issued at the national level, which would facilitate the task of provincial coordination as well as the health zones involved in the integration process. Task sharing between the stakeholders involved and strong leadership from decision-makers and managers at all levels of the system is then conducted. (P15, coordinator, national mental health program)*

##### Human resources

Human resources. One interviewee (P17, staff, development partners) noted that if PSPs become aware of their lack of knowledge in dealing with mental health issues and begin to express a desire to address this gap, mental health integration will be promoted. This finding supports the idea of organizing in-service training on mental health and managing mental health problems.

One of the healthcare providers interviewed said:

*Collaboration between specialized hospital providers, health center teams, and family members will facilitate integration, especially if they collaborate around the mental patient’s care project. (P3, healthcare provider, primary care service)*

For an interviewee, the presence of a mentor or coach who is a mental health professional is a factor that facilitates integration since this specialist will ensure the mentoring/coaching of PCPs in the care of people living experience of mental health conditions. He said:

*The presence of a full-time mental health specialist in primary care settings is a crucial facilitator because it helps nonspecialists adapt to caring for the mentally ill. (P4, manager, health district)*

##### Financing

When asked about health financing-related facilitators, one participant indicated that specific financial support from technical and financial partners or the state budget would enable the integration process to run smoothly. He said:

*The existence of financial support from development partners and/or the opening of a separate budget line dedicated to the integration of mental health into the budget allocated to the Ministry of Public Health and financial incentives for healthcare providers to participate in patient care will facilitate successful integration. (P13, coordinator, national mental health program)*

##### Medication

A respondent insisted on clarifying the supply circuit for psychotropic drugs to enable health structures that have integrated mental health to be served whenever needed. He said:

*If we improve the supply circuit for essential generic medicines, this will be a facilitator, as it will enable a regular supply of psychotropic medicines to primary care facilities. (P3, healthcare provider, primary care service)*

##### Service delivery

For this block, the KI explained that the fact that healthcare providers recognize mental health problems as a public health issue is an important facilitator of integration. He said:

*As soon as healthcare providers agree that mental disorders are common and public health problems, they decide to address them and adopt positive attitudes toward the provision of mental health care. This is, in my opinion, a factor favoring integration. (P8, implementer, national NGO)*

The other respondent stressed that once stakeholders agree on the benefits of offering mental health care to patients in primary care settings, this is a factor that promotes integration. From their statements, we noted the following:

*Supporting the idea of providing mental health care within the health center and at the general referral hospital is a sign of successful integration on the part of healthcare providers. These patients will require the availability of management protocols to improve the quality of care. (P19, policymaker, ministry of public health)*

##### Infrastructure

Regarding infrastructure-related facilitators, one KI stressed that for integration to be successful, there is a need for health infrastructures with a dedicated setting for consultation and mental health care. He put it as follows:

*The existence of a workspace dedicated to the consultation, observation, and care of people living experience of mental health conditions. (P1, healthcare provider, primary care service)*

##### Information

A participant indicated that, for integration to be successful, it is necessary to have (i) a clearly defined list of indicators, (ii) data collection tools available in health facilities, and (iii) health facilities set up in DHIS2 (i.e., District Health Information System version 2). He stated:

*It is important for the PNSM to work with the National Health Information System (NHIS) team to clearly define mental health indicators, develop data collection tools, and make them available and for health structures that have integrated mental health to be connected to the DHIS2. (P25, staff, development partners)*

### Health system barriers

#### Governance

KI mentioned that the top-down approach, bureaucratization, lack of formalization of relations between managers and healthcare providers, centralized administration, and failure to comply with norms are governance-related barriers to the successful integration of mental health. He stated:

*The top-down approach applied in the design of some integration programs and the bureaucratization of implementation management, as well as the lack of formalization of relationships between (mental) health managers and healthcare providers, are likely to make integration difficult. Similarly, centralization of service administration and noncompliance with standards are obstacles to the integration of mental health into the primary care system. (P24, policymaker, ministry of public health)*

The lack of priority given to integrating mental health from central to peripheral levels and the consequent inability to allocate substantial resources to mental health were seen as barriers. One interviewee highlighted the following:

*The fact that mental health care is not prioritized from the central level of the Ministry of Health down to the health districts is that programs to integrate mental health care are lacking. It is not enough to have an ambitious national mental health policy. Prioritizing integration also means making available substantial financial, material, and human resources. (P14, coordinator, national mental health program)*

In the same vein, a participant highlighted the lack of clarity in the formulation of integration objectives, the lack of collaboration between different categories of providers, and poor team coordination as obstacles to the implementation of the integration strategy. He stated as follows:

*The lack of clearly expressed mental health integration objectives will not allow the peripheral level (health districts) to implement integration activities. In addition, the limited collaboration between Western medical care services, traditional care structures, and churches providing spiritual support is proving to be a barrier to integration in our environment, where cultural and spiritual beliefs are deeply rooted in the population. In addition, inadequate coordination between PCPs such as general practitioners and mental health specialists will not allow the integration experience to be successful. (P3, healthcare provider, primary care service)*

#### Human resources

Turning to workforce-related barriers, an informant highlighted the shortage of (mental) health professionals:

*[…] The quantitative shortage of mental health specialists (psychiatrists, psychologists, mental health nurses, etc.) and the qualitative shortage of generalist providers (primary care physicians, nurse practitioners, etc.) are major obstacles to integrating mental health into primary care. (P11, coordinator, national mental health program)*

For his part, an informant revealed a barrier related to sporadic visits to specialists designated to provide coaching to PCPs engaged in implementing integration. He stated as follows:

*Irregular visits by the psychiatrist assigned to coach or mentor the primary care team can create major disruptions in the integration process. (P3, healthcare provider, primary care service)*

#### Medicines and technologies

Barriers related to this building block were identified. In an interview, a KI indicated that the lack of medicines in primary care facilities would hamper the implementation of integration. From his statements, we retained the following:

*The lack of appropriate psychotropic drugs to treat mental illness in healthcare facilities in health districts is an obstacle that prevents these structures from retaining the gains of integration. (P31, implementer, national NGO)*

Another participant insisted on a lack of clear definition of the supply circuit for psychotropic drugs and on certain restrictions prohibiting nurses in health centers from prescribing neuroleptics. He explained:

*If we have not clearly defined the supply circuit for psychotropic drugs, we will have problems with stock-outs and drug management. In addition, an irregular supply of mental health drugs, which are on the national essential drug list, and a restriction on the prescription of psychotropic drugs by nurses in health centers are obstacles because they will make things rather complicated in the field. (P3, healthcare provider, primary care service)*

In addition, the lack of diagnostic tools was highlighted as an obstacle to supply. A KI put it this way:

*Patient screening, treatment, and follow-up tools such as appointment books, if absent from healthcare facilities or if they are too long or poorly designed to be integrated into practice, will disrupt care activities. (P4, manager, health district)*

#### Financing

Based on the KIs’ statements, financing-related barriers were explored. First, lack of funding was mentioned as a major barrier to implementing mental health integration. A KI interviewed said,

*Despite our intellectual efforts to design clear guidelines for mental health integration into primary healthcare, the lack of funding for the mental health subsector poses significant challenges to the successful integration of mental health into primary healthcare in the country. (P22, policymaker, ministry of public health)*

According to one respondent, the lack of alignment of technical and financial partners with the (local) health system development plan is one of the obstacles to mental health integration activities. He put it this way:

*Yes, the fact that the financial partners who support the Ministry of Health are not aligned with the country’s health priorities in a context where the state budget is very insufficient is a major obstacle to the implementation of projects to integrate mental health into PHC. (P21, coordinator, national mental health program)*

#### Service delivery

The verbatims extracted from the data show that barriers concern both members of the care team and service users. An informant mentioned that healthcare providers feel that they do not currently have the time needed for mental health care. He stated that.

*The perception that PCPs lack the time to provide mental health care and that they devote limited time to each patient is a barrier to successful integration when considering the complex needs of patients with psychiatric problems. (P2, manager, health district)*

One participant noted that when healthcare providers think that treating mental illness is the prerogative of specialists, this may be a barrier to providing mental health care in primary care services. He stated:

*To date, healthcare providers are convinced that the mentally ill should not be treated in health centers and that treating the mentally ill in primary care would put other patients at risk. (P13, coordinator, national mental health program)*

An informant added an exclusionary barrier for some mental health workers, stating that.

*The exclusion from the care process of other caregivers who are not trained in the Western medical model, e.g., traditional healers and spiritual healers, while patients receive some nonmedical care is a barrier that is likely to affect the quality of provision. (P9, community leader, health area)*

On the other hand, patients’ dependence on traditional therapy services may constitute an obstacle to the provision of services in health facilities that integrate mental health. The informants’ statements revealed the following:

*Another obstacle to the provision of mental health services in facilities that have integrated the mental health component is that patients most often turn to traditional healers or religious healers rather than to modern health facilities. (P3, healthcare provider, primary care service)*

#### Infrastructure

An interviewee indicated that a very limited number of infrastructural resources, particularly the physical space of health centers, is a barrier likely to disrupt the progress of integration activities. He stated it as follows:

*The lack of examination, care, and accommodation space for patients in our health centers and general referral hospitals is an obstacle to integration because it makes it difficult to conduct such activities. In addition, the narrowness of health facilities affects the physical distance between agitated and calm patients. (P3, healthcare provider, primary care service)*

#### Information

An informant noted that most district health services (e.g., health centers) involved in the mental health integration process do not have access to the DHIS2 mental health module to manage the data they generate, with the exception of health district officers in South and North Kivu who have received training. He highlighted this as a major barrier to sharing mental health information. In her statement, we noted the following:

*Mental health data collection tools are currently lacking in some health facilities, and these facilities are not even configured for DHIS2. This is a barrier to integration because attending nurses and nurse supervisors cannot communicate information to the central level. (P6, manager, health district)*

An interviewee explained that if people do not know that a new mental health care offer has been added to district health services, this would constitute a barrier. She stated that.

*The fact that people—both healthcare providers and the general public—are unaware of the availability of mental health care that has been integrated into health centers and general hospitals is a major obstacle […]. (P11, coordinator, national mental health program)*

Table 2 summarizes the factors explored in the interviews that are likely to facilitate or hinder successful mental health integration, grouped by health system building block, stakeholder concerned, and health system level.

**Table 2:** Facilitators and barriers to integration according to health system building blocks, stakeholders, and system levels

Facilitators	For whom?	At what HS level?	Barriers	For whom?	At what HS level?
Governance					
Existence of a mental health service coverage plan	Policymakers and managers	⦿	Top-down Approach to Integration Program Design	Managers and healthcare providers	⨀⚈
Commitment of managers	Managers	⦿	Bureaucratization	Healthcare providers	⚈
Guidelines for mental health integration	Managers	⦿	Lack of formalization of the relations between managers and providers	Healthcare providers	⨀⚈
Mental health task sharing	Managers and healthcare providers	⦿⚈	Centralized administration	Healthcare providers	⨀⚈
Strong leadership	Policymakers and managers	⦿	Failure to comply with norms	Policymakers and managers	⨀⚈
			Lack of separation of responsibilities among actors	Managers, implementers, providers, and service users	⦿⨀⚈
			Lack of support for health district managers	Managers	⚈
			Lack of priority given to integrating mental health	Policymakers and managers	⦿⨀⚈
			Poor time management	Policymakers and managers	⦿⨀⚈
			Lack of clarity in developing integration goals	Managers and implementers	⨀⚈
**Human resources**					
Becoming aware of a lack of knowledge	Healthcare providers	⚈	Shortage of (mental) health professionals	Managers	⨀⚈
(Prior) training in mental health	Healthcare providers	⚈	Sporadic visits by specialists selected to provide coaching to PCPs	Healthcare providers	⚈
Collaboration between specialized and nonspecialized providers	Healthcare providers	⚈	Insufficient knowledge to diagnose mental health problems	Healthcare providers	⚈
Presence of a mentor or coach	Healthcare providers	⚈			
**Financing**					
Dedicated financial support for integration from technical and financial partners	Managers and implementers	⦿⨀⚈	Lack of sustainable funding for implementing mental health integration	Managers and implementers	⦿⨀⚈
Separate budget line dedicated to integration	Policymakers, managers, and implementers	⦿⨀⚈	Lack of alignment of technical and financial partners with the (local) health system development plan	Managers and implementers	⦿⨀⚈
Financial motivation of providers	Healthcare providers	⚈	Low financial motivation of mental health specialists	Healthcare providers	⚈
			Inadequate budgets and inequitable budget allocation	Managers and implementers	⦿⨀⚈
**Medicines and technology**					
Permanent presence of medication	Managers, implementers, and providers	⨀⚈	Lack of medicines in primary care facilities	Healthcare providers and service users	⨀⚈
Clear supply circuit for psychotropic drugs	Managers, implementers, and healthcare providers	⨀⚈	Lack of a clear definition of the supply circuit for psychotropic drugs	Managers, implementers, and providers	⨀⚈
Delegating the task of prescribing psychotropic drugs to PCPs	Healthcare providers	⚈	Restrictions prohibiting PCPs from prescribing neuroleptics	Healthcare providers	⚈
			Absence of a means of transport	Service users	⚈
			Lack of diagnostic tools	Healthcare providers	⚈
**Service delivery**					
Recognition of mental health problems as a public health issue	Implementers	⨀⚈	Feeling of not having enough time for mental health care	Implementers	⚈
Agree on the benefits of offering patients mental health care	Implementers	⨀⚈	Thinking that mental illness treatment is the prerogative of specialists	Implementers and healthcare providers	⚈
Positive attitudes toward the provision of mental health care	Implementers and providers	⨀⚈	Uncertain of his/her role as a mental health care provider	Implementers and healthcare providers	⚈
Care protocols available	Healthcare providers	⚈	Thinking that mental disorders are difficult to diagnose	Implementers and healthcare providers	⚈
			Exclusion from the care process of other caregivers (e.g., traditional healers)	Healthcare providers	⚈
			Believe in the ineffectiveness of modern health care	Implementers and providers	⚈
			Exclusive dependence on traditional therapy services	Healthcare providers	⚈
**Infrastructure**					
Dedicated location for consultation and mental health care	Healthcare providers	⚈	Lack of examination, care, and accommodation space for patients	Healthcare providers	⚈
			Narrowness of space, leading to noncompliance with the physical distance rule	Healthcare providers	⚈
**Information**					
Clearly defined list of indicators	Managers	⨀⚈	Refusal to have information documented	Healthcare providers	⚈
Health facilities set up in DHIS2	Managers	⨀⚈	Lack of parameterization of primary care facilities in DHIS2	Managers	⦿⨀⚈
Data collection tools available in health facilities	Healthcare providers	⚈	Lack of knowledge about the new mental health care offer added to primary care facilities	Population	⚈

HS, health system; ⦿ = central level; ⨀ = provincial level; ⚈ = local level; DHIS2: District Health Information System – version 2; PCPs: Primary care providers

### Facilitators and barriers at the community level

#### Facilitators

One participant noted that community involvement in activities facilitates integration because it influences changes in attitudes toward stigmatization and other violations. He said:

*Community involvement in integration activities and other social strata will facilitate the reduction of stigmatization, discrimination, and other forms of violence against the mentally ill, which will encourage them to seek treatment. (P10, community leader, health area)*

According to one respondent, the fact that the community now recognizes that shackling people living experience of mental health conditions is a bad practice is a factor that facilitates integration. He stated:

*The fact that some community members already recognize that handcuffing violent people living experience of mental health conditions and/or putting them in solitary confinement is a violation of human rights and that it would be better to hand them over to a career for proper care is proving to be a success factor for integration. However, it is best to keep working hard to change the mentality of the entire community. (P12, household head, health area)*

A community respondent mentioned that free mental health care would make it easier to sustain integration activities. His statements are reproduced below:

*It is essential to make mental health care free, including medication; this will facilitate the success and maintenance of training. (P12, household head, health area)*

#### Barriers

An informant stated that stigma within the community and fear of lack of confidentiality among healthcare providers are barriers to accessing health facilities that integrate mental health services. He said:

*The main barriers at the population level are fear of stigma related to the mental illness they suffer from and the fact that confidentiality needs are not met when they go to the hospital. (P26, implementer, international NGO)*

Therefore, the participant talked about awareness, saying:

*A lack of mental health awareness means that the knowledge and information people have is wrong […]. If people do not change their mental health by raising awareness, this will be an obstacle to the acceptability of mental health care and even to its use in the facilities that have integrated it. (P30, implementer, national NGO)*

### Quantitative findings

As shown in Table 3, the explanatory variables were described by estimating the frequencies of responses to ‘agree’ and ‘disagree’ that these factors could be facilitators or barriers to integration and then expressing them as percentages.

**Table 3:** Frequency with which participants agreed with health system factors likely to facilitate/impede integration (*N* = 413)

Facilitators by building blocks	*n* (%)		Barriers by building blocks	*n* (%)
Governance				
Health managers’ commitment to integration	301(72.9)		Lack of mental health priority	113(27.4)
Task sharing among stakeholders	218(52.8)		Complex programs to implement	290(70.2)
Presence of a mental health service coverage plan	98(23.7)		Lack of mental health referent	96(23.2)
Accessible integration guidelines	112(27.1)		Top-down Approach to Integration Program Design	103(24.9)
			Lack of clear national guidelines for integration	107(25.9)
Human resources				
Sufficient number of nonspecialist providers	299(72.4)		Lack of specialists to mentor PCPs	246(59.6)
Availability of specialists to coach PCPs	221(53.5)		Low retention rate of trained of health professionals	306(74.1)
Presence of multidisciplinary teams	73(17.7)		Fear of misdiagnosis	54(13.1)
			Sporadic visits by specialists to mentor PCPs	62(15.0)
Medicines and technologies				
Sustainable Drug Supply	202(48.9)		Lack of psychotropic medicines	211(51.1)
Simple treatment regimen	197(47.7)		Complex treatment regimens	31(7.5)
Dedicated mental health workstation	154(37.3)		Lack of mental health equipment	64(15.5)
			Lack of Diagnostic and Treatment Guidelines	99(24.0)
			Absence of a means of transport	94(22.8)
Service delivery				
Primary care services prepared for integration	49(11.9)		Weakness of public health facilities	269(65.1)
Positive attitudes toward the provision of mental health care	42(10.2)		Beliefs linked to the efficacy of traditional medicine	280(67.8)
			Inability to diagnose and treat	65(15.7)
			Exclusion of other caregivers from the care process	55(13.3)
Financing				
Existence of financial support from development partners	218(52.8)		Lack of funding for integration	232(56.2)
Expectations of free mental health care	308(74.6)		Belief that an out-of-pocket payment will be applied	275(66.6)
Financial incentives for healthcare providers	294(71.2)		Cost of recruiting new mental health specialists	109(26.4)
			Low financial motivation of specialists	86(20.8)
Information				
Existence of mental health indicators in the NHIS	209(50.6)		Lack of reporting tools	277(67.1)
Health services connected to DHIS2	71(17.2)		Lack of a mental health connection to DHIS2	199(48.2)
Clearly defined list of indicators	49(11.9)			
Infrastructure				
Extension of existing healthcare facilities	331(80.1)		Narrowness Health Centers and District Hospitals	266(64.4)
Building new psychiatric hospitals	198(47.9)		Private ownership of many health facilities	230(55.7)
			No dedicated mental health consultation room	201(48.7)

PCPs, primary care providers; NHIS, national health information system; DHIS2, district health information system – version 2

From the multivariate analysis of facilitators, we found a total of nine factors that were at least 1 time more likely to facilitate integration when comparing the two groups that agreed or disagreed that integration was possible. The following three of these facilitators were very predominant: task sharing among stakeholders (aOR: 3.48, 95% CI: 2.49–4.84), sufficient nonspecialist healthcare providers (aOR: 1.41, 95% CI: 1.25–1.70) and the existence of mental health indicators in the NHIS (aOR: 2.12, 95% CI: 2.07–2.22) (Table 4).

**Table 4:** Health system facilitators for the integration of mental health (*n* = 413)

Predictors by building blocks	*n*	Wald-χ^2^	aOR	95%CI	
				Lower	Upper
Governance					
Health managers’ commitment to integration	301	6.89^**^	2.56	2.37	2.85
Task sharing among stakeholders	218	19.01^***^	3.48	2.49	4.84
Mental health integration guidelines	124	4.61^*^	1.62	1.41	1.92
Human resources					
Sufficient number of nonspecialist providers	299	18.86^***^	1.41	1.25	1.70
Availability of specialists to mentor primary care providers	221	5.05^***^	1.36	1.22	1.58
Medicines and technologies					
Sustainable Drug Supply	202	9.71^**^	2.15	2.04	2.49
Financing					
Existence of financial support from development partners	218	3.91^*^	2.01	1.21	4.05
Information					
Existence of mental health indicators in the NHIS	209	19.36^***^	2.12	2.07	2.22
Infrastructure					
Extension of existing healthcare facilities	331	8.71^**^	2.57	1.11	5.93

Dependent variable: Integration of mental health (possible/not possible); aOR: adjusted odd ratio; NHIS, national health information system; CI, confidence interval; *: significant at *p* < 0.05; **: significant at *p* < 0.01; ***: significant at *p* < 0.001

From the multivariate analysis of barriers, we identified 12 factors that were at least 1 time more likely to impede integration when comparing the two groups that agreed or disagreed that integration was possible. The following four of these facilitators were very predominant: lack of clear guidelines for mental health integration (aOR: 12.03, 95% CI: 6.41–17.66), lack of specialists (aOR: 8.04, 95% CI: 5.11–11.13), beliefs were linked to the efficacy of traditional medicine (aOR: 13.02, 95% CI: 7.62–18.42), and lack of funding for integration (aOR: 7.02, 95% CI: 4.41–10.23) (Table 5).

**Table 5:** Health system barriers to the integration of mental health (*N* = 413)

Predictors by building blocks	*n*	Wald-χ^2^	aOR	95%CI	
				Lower	Upper
Governance					
Lack of clear guidelines for mental health integration	107	129.20^***^	12.03	6.41	17.66
Lack of mental health priority	113	3.91^*^	1.74	1.22	1.96
Lack of mental health referent	96	5.48^*^	1.62	1.11	2.32
Human resources					
Lack of specialists to mentor primary care providers	246	601.38^***^	8.04	5.11	11.13
Low rate of retention of the current care staff	306	7.16^**^	1.82	1.62	1.98
Medicines and technologies					
Lack of psychotropic medicines	211	40.10^***^	3.06	2.71	4.05
Service delivery					
Beliefs linked to the efficacy of traditional medicine	280	562.07^***^	13.02	7.62	18.42
Financing					
Lack of funding for integration	232	318.18^***^	7.02	4.41	10.23
Belief that an out-of-pocket payment will be applied	275	5.02^*^	1.80	1.42	1.97
Information					
Lack of reporting tools	277	40.19^***^	3.56	1.71	5.95
Infrastructure					
Narrowness Health Centers and District Hospitals	266	5.48^*^	1.37	1.09	1.58
Private ownership of several health facilities	230	13.91^***^	2.15	2.01	2.21

Dependent variable: Integration of mental health (possible/not possible); aOR: adjusted odd ratio; CI, confidence interval; *: significant at *p* < 0.05; **: significant at *p* < 0.01; ***: significant at *p* < 0.001

## Discussion

In this study, we sought to identify health system factors that are perceived by various stakeholders as facilitating or hindering the integration of mental health services into primary care settings. The findings highlighted key health system facilitators of this integration that should be consolidated. They also revealed some health system barriers that need to be addressed to increase the success rates of mental health integration programs in the DRC and other LMICs, aiming to fill treatment gaps by (re)organizing primary mental health care in nonspecialized health care settings.

Primary mental health care provided in nonspecialized care settings plays an essential role in addressing mental health problems. Given that these primary care settings are the first point of contact for many people in need, successfully integrating mental health into primary care requires consolidating, in all building blocks, the health system factors that enable this integration and tackling those that hinder it.

### Health system facilitators of integration

Governance can be a driver of success in HSS initiatives, such as the integration of mental health into primary care. Even if there is a good policy framework to support this integration, it may not be sufficient to ensure a real transformation of the health system toward integrated primary mental health care if there is not good governance to ensure the implementation of the policy requirements [36]. In this regard, our findings, based on participants’ statements, showed that the presence of a mental health care coverage plan, strong management commitment to integration (*p* < 0.01), the presence of national guidelines for mental health integration, task sharing among stakeholders (*p* < 0.001), and strong management leadership were the key governance factors likely to promote successful integration.

Despite the recognition of its key role in the success of integration, the governance of the mental health system in the DRC remains very weak, relying solely on the commitment and leadership of PNSM managers. Admittedly, the DRC has a stand-alone mental health policy and human resources that are allocated to its implementation, albeit inequitably distributed [2]. However, the PNSM currently lacks a validated mental health care plan, a clear description of the roles of actors involved in integration, and national guidelines for mental health integration. It is important that the current drafting of the national multisectoral mental health strategy be accompanied by these documents, which will help strengthen the governance of the mental health system.

Health professionals are recognized as key resources for the success of health interventions. Our findings indicate that PCPs’ awareness of their lack of mental health knowledge, collaboration between specialist and nonspecialist providers, sufficient nonspecialist providers (*p* < 0.001), and the availability of specialists to mentor PCPs (*p* < 0.001) are factors that may facilitate integration. At present, the main human resource challenge is that the DRC currently has a total of 0.9 MHWs per 100,000 inhabitants [27]. These MHWs are inequitably distributed across the country, with the vast majority working in cities. To solve these human resource problems, we need to develop a production plan for mental health service providers, create a framework for interministerial collaboration between higher education and public health, recruit staff on the basis of their qualifications and skills, consider the needs of users, and organize on-the-job training in accordance with expressed needs.

Financial support dedicated to integration from technical and financial partners, a separate budget line dedicated to integration, the financial motivation of providers, and free mental health care have been declared enablers. To consolidate these facilitators, development partners must align themselves with the subsectoral mental health policy and health development plan of health districts and provincial health divisions, which are considered local priorities. Funding must be comprehensive and structural. However, according to the WHO [23], health financing refers to the mobilization, pooling, and allocation of financial resources to meet the health needs of populations, either individually or collectively. Today, we know that the goal of health financing is to ensure UHC or affordable access to care, including primary mental health care, for all people. For financing to be an enabler of mental health integration, the three essential functions of financing (fundraising, pooling and purchasing) must function successfully. To achieve UHC, including primary mental health care with free care for the indigent, it is important to introduce progressive mandatory contributions (i.e., the rich pay a relatively larger share of their income than the poor) [37] and to pool resources at the highest possible level to maximize efficiency and redistribution. We propose to clearly define a package of services that includes social assistance, unemployment/economic opportunity costs, curative mental health care, and social reintegration activities. Free mental health care should then be used as a means to improve financial access to mental health care for direct service users, i.e., patients who are often indigent.

Medicines are a way that patients are attracted to healthcare facilities, especially when they believe they will cure them [38]. Therefore, it is one of its health system building blocks for medicines and technologies [23]. Our results show that the permanent presence of medicines in health facilities, a clear supply chain for psychotropic medicines, a sustainable supply of medicines, and the delegation of the task of prescribing psychotropic medicines to PHC providers are likely to have facilitated integration. For these facilitators to contribute to the availability of essential generic psychotropic medicines, the PNSM should update and make available the list of these psychotropic medicines. Training drug management stakeholders, particularly at the health district level, and advocacy with the government and technical and financial partners supporting integration programs are promising avenues.

Recognition of mental health problems as a public health issue, PCPs’ agreement on the benefits of providing mental health care to patients, PCPs’ positive attitudes toward providing mental health care in primary care settings, and the availability of psychosocial and mental health care protocols in primary care settings were identified as service delivery factors likely to facilitate integration. For these facilitators to provide opportunities for integration in LMIC settings where mental health facilities are severely lacking, it is necessary to focus on primary care services. However, to ensure that people benefit from mental health care that is/will be integrated into primary care structures, it is important to increase awareness to increase mental health knowledge and improve mental health care-seeking behavior [39]. This includes building the capacity of PCPs by organizing theoretical and practical training to help them acquire basic theoretical knowledge about mental health and skills to address mental health problems. The WHO mhGAP training manuals [40] can be adapted using a modular approach that considers the priorities and specificity of each context.

In the DRC, the stigmatization of people living experience of severe mental health conditions appears to be a factor in their exclusion from mental health care, which further reduces their access to care in health facilities. These people are often seen as ‘cursed’ with no possibility of recovery. Children with behavioral disorders who are stigmatized are sometimes considered ‘witch children’ or ‘cursed’, which discourages them from seeking treatment [41]. Because people living experience of severe mental health conditions are always confronted with stigmatizing attitudes, anti-stigma campaigns at the community level and in care facilities, as well as reception and service provision in appropriate infrastructures, would be highly necessary. Hence, in our study, respondents indicated that the existence of spaces dedicated to mental health counseling and care and the expansion of existing health care facilities are likely to facilitate integration. The DRC has only 6 psychiatric hospitals, 1 inpatient mental health service, 6 community/nonhospital outpatient mental health services, and 27 other outpatient services (e.g., day care or mental health treatment services) for 100 million inhabitants spread over an area of 2,345,000 km^2^ [2], which represents a very limited capacity. Therefore, there is an urgent need to invest in the construction of new mental health facilities, but above all, to improve the capacity of primary care structures into which mental health is/will be integrated.

### Health system barriers to integration

Our findings revealed some governance-related problems perceived as barriers to integration. These included top-down program design, poor coordination of activities with a lack of support for district managers, lack of priority given to mental health integration, lack of clear national guidelines for mental health integration (*p* < 0.001), and lack of a mental health referent. In practice, in resource-limited countries such as the DRC, the top-down approach to designing targeted health interventions is often adopted when it is believed, rightly or wrongly, that the operational level of the health system has nothing to offer in terms of funding or ideas. As a result, there is often a lack of ownership and sustainability of interventions once funding ceases. We should adopt a bottom-up, participatory approach that values indigenous knowledge because the management of mental health issues is strongly linked to cultural beliefs. The lack of a mental health referent may be linked to a shortage of mental health professionals. To alleviate this situation, it may be necessary to designate a member of the care team who appears to have special skills and an interest in mental health issues and who can interact with his or her colleagues. For this to work, a redefinition of his or her workload within the health facility management team would need to be reconsidered. Given the lack of national integration guidelines and the fact that PCPs have limited knowledge and skills in using guidelines and treating mental health problems, this could affect the integration process [20]. It is possible that there are different local guidelines for mental health integration in the DRC, developed by health districts that have integrated mental health into their primary care settings. Therefore, it is necessary for the DRC PNSM to harmonize, adopt, and make available national guidelines for integrating mental health into primary health care to standardize the approach and facilitate work at the health district level.

It is important to note that these results corroborate those of a situation analysis carried out by the DRC PNSM in 2022, as mentioned in its operational action plan for 2023, which indicates that the governance of the mental health system is characterized by several problems. These include poor coordination of mental health interventions, a lack of normative documents, and weak accountability in the transmission of instructions and information. To address these weaknesses, leadership needs to be strengthened through on-the-job training, inspection, monitoring, and supervision organized by decision-makers in the Ministry of Health.

Our findings showed that a shortage of mental health professionals, sporadic visits by specialists selected to coach PCPs, inadequate knowledge to diagnose mental health problems, and the low retention rate of trained PCPs were key barriers to integration. There is evidence of a shortage of mental health professionals in most LMICs. There is evidence of a shortage of mental health professionals in most LMICs. To fill this gap, mental health care should be provided as part of primary care through strategies such as task sharing with nonspecialist providers, family caregivers, and other health workers [42, 43]. In this way, mental health professionals could continue to train, supervise, and mentor nonspecialist providers to whom mental health tasks have been delegated. The Ministry of Health, through its PNSM, should ensure effective leadership and management of the available mental health workforce by implementing key strategies such as recruitment, retention, and equitable distribution [43]. In the meantime, the ministries of health and higher education should work together to develop a plan for the initial training of mental health professionals.

Mental health financing may be one of the weakest links in the chain of integration in resource-limited countries. Our analysis showed that the lack of a government budget allocated to integration (*p* < 0.05) and the belief that mental health care would be paid out-of-pocket by service users once integration was achieved (*p* < 0.001) were identified as factors that may hinder integration. In contrast, the presence of financial support from development partners (*p* < 0.05) was identified as a potential facilitator of integration. To facilitate the alignment of technical and financial partners with national priorities (e.g., mental health integration), it was recommended to improve the health financing environment by strengthening leadership, accountability, transparency, and equity among health managers; avoiding stifling them with top-down approaches to agreements with these partners; and implementing a truly consensual sectoral approach around a single plan, synergistic financing, and a single monitoring and evaluation system [44].

Our results showed that the lack of psychotropic medicines in primary care facilities and the absence of a clearly defined supply circuit for psychotropic drugs are factors likely to hinder integration. In a study conducted in seven LMICs, including the DRC, researchers [45] found that mental health medicines (e.g., amitriptyline) were virtually unavailable in health facilities, particularly in public primary care settings. They emphasized that the limited availability of essential psychotropic medicines could limit efforts to integrate mental health services into primary care in LMICs and called for the development of supply, distribution, and capacity building in the appropriate use of essential mental health medicines in these countries. When integrating mental health into primary care, program managers and implementers must plan optimal strategies for medication supply and recycling. This can help prevent stock-outs that could negatively affect patient treatment.

In provinces where integration has begun, service delivery barriers have been identified. These include a lack of diagnostic tools, the belief that specialists are solely responsible for mental health care, the perception that mental disorders are difficult to diagnose and treat, the exclusion of other providers (e.g., traditional healers) from the care process, and beliefs about the effectiveness of traditional medicine (*p* < 0.001). In its Operational Action Plan 2023, the PNSM mentioned the following delivery-related problems: low (geographical) coverage of mental health services and low quality of care and services. This may be due, in particular, to the low proportion of providers who have received in-service training in mental health and to the lack of supervision and/or nonsupervision of primary care facilities. It is essential to encourage mental health workers to work primarily in multidisciplinary teams and to promote collaborative approaches to care [15].

In terms of health infrastructure, our findings showed that the lack of examination, treatment, and accommodation space for patients, the cramped conditions of health centers and district hospitals that resulted in noncompliance with the physical distancing rule, and the private ownership of several health facilities were barriers to integration. A study conducted in South Africa [36] revealed that inadequate quantity and quality of existing health infrastructure, lack of coordinated infrastructure planning between the sectors involved, and lack of adequate consultation space in primary care facilities were among the factors hindering integration. The authors recommend the provision of adequate mental health consultation space in primary care facilities that integrate mental health care.

The Congolese Ministry of Health’s DHIS2 includes about a dozen mental health indicators in one of the modules of the mental health component of the National Health Information System (NHIS). However, only primary care facilities in the two provincial health divisions of South Kivu and North Kivu are currently computing these indicators, since they were chosen to serve pilot areas following a programmatic decision by the Ministry of Public Health. The fact that the majority of primary care facilities that have integrated mental health activities are not doing so jeopardizes full data reporting by care providers. In this context, it is not possible to fully appreciate the extent of mental health problems, nor is possible to properly assess the outcomes of integration of mental health in terms of health service utilization. Health information is one of the poorest performing building blocks of the mental health system in the DRC. The current availability of mental health data is very low, and mental health reporting and research are still in a rudimentary phase. The few data that are available are generally limited to general statistics. In 2020, only 6 research articles on mental health were published, representing a dismal 2.14% of the country’s total research output and 1.0% of the total mental health research output in the WHO African Region [2]. Our findings confirm field observations of a lack of harmonized data collection tools at all levels and the use of nonstandardized reporting templates in health districts that have integrated mental health, as well as the unavailability of manuals on how to complete the tools. To improve this situation, it is necessary to provide harmonized data collection tools and train staff in the use of DHIS2 software. This is feasible with the involvement of policy makers in the Ministry of Public Health.

### Strengths and limitations

This study has three strengths. The first is the multimethod design, which allowed us to use both qualitative and quantitative approaches. Using this design, we identified key factors that need to be considered for the successful integration of mental health services into primary care. The second is related to the WHO building blocks framework, which is currently widely used in health system research [46]. Using this WHO framework as a reference, this study captured the main health system factors affecting mental health integration from a more systemic and comprehensive perspective. It seems very relevant to further expand and finetune this conceptual framework and make it more fit to the specific African context, for instance in enabling health system managers and researchers to analyze the dimensions of equity, societal norms and social exclusion, in the various building blocks and/or functions of the health system. But also, in encouraging researchers and practitioners to address aspects of communication that take into account specific beliefs, habits and customs strongly related to (mental) health in the African context. Our findings complement those of previous studies that have used other frameworks, focusing mainly on constraints such as accessibility of care, patient flow processes, health facilities, human resources, and gender factors, essentially from the perspective of PCPs [20, 47]. The third pertains to the fact that the study findings reflect the views of a range of stakeholders involved in mental health integration, such as the Ministry of Health decision-makers, program managers, implementers, technical and financial partners, mental health specialists, PCPs, and service users.

However, this study has three limitations. First, this study used an exploratory cross-sectional design to identify facilitators of and barriers to mental health integration based on stakeholders’ perceptions and opinions. Because perceptions and opinions are subjective, these findings may help to replicate this study using other methodological approaches, such as (quasi)experimental designs, to establish cause-and-effect relationships. Second, the statistical regression model included several explanatory variables. Other variables could not be included in the model because their frequencies were zero. However, because the data covered all of the building blocks in the analysis, these results can be essential for making evidence-based decisions about integration. Third, although the service users were assisted by the interviewers in completing the questionnaires or in giving further explanations during the interviews, we are very well aware that the questionnaires and interview guides were quite complex. They indeed required a minimal level of understanding of health systems which may have been challenging for community members and service users. To minimize response artifacts, researchers assisted participants in completing the questionnaires and provided clarification, and we triangulated the data provided by these service users with those provided by key informants. However, this was only a partial solution, as community members and service users may be reluctant to ask questions or express difficulty in understanding certain (very common) concepts. This limitation needs to be addressed in future studies.

## Conclusions

This study provides helpful insights into a complex area that requires significant investment to address major gaps and inequities in access to mental health care. The results show that integrating mental health into primary care is feasible but that various health system barriers, such as lack of funding and stigma, remain major challenges. To mobilize sufficient financial resources for integration, it is important that mental health be effectively included in UHC and that policymakers show a clear political will to invest in mental health and psychosocial support. In addition, a dialog is needed to explore ways to help governments invest more in mental health, particularly in the integration of mental health services into primary care settings. To achieve this goal, a consortium should be established that brings together all national and international health system stakeholders active in the field of mental health to develop a common vision for integration and to support fundraising efforts.

To address the challenge of stigma, anti-stigma campaigns involving various actors involved in the supply and demand of mental health care, if well implemented, will lead to a change in attitudes toward mental health and people living experience of mental health conditions, which in turn will lead to improved uptake of care by all patients, including those with mental health problems. To ensure the effectiveness of these anti-stigma interventions, a short-term quasiexperimental study is planned to test, for example, the effectiveness of awareness-raising and in-service training interventions in reducing mental health-related stigma and improving care seeking among patients, including those with mental health problems. Finally, we hope that our findings will inform the development of national strategies and guidelines for the integration of mental health into primary care to improve progress toward UHC, equitable access to quality healthcare services, and the reduction of mental health disparities in the DRC and other LMICs.

### Electronic supplementary material

Below is the link to the electronic supplementary material.

Additional file 1: Text S1. Interview guide

Additional file 2: Text S2. Individual survey questionnaire

## Data Availability

Data are available upon request from the corresponding author.

## Abbreviations

CHW: Community health worker
DALYs: Disability-adjusted life years
DGD: Belgian Directorate General for Development Cooperation
DHIS2: District Health Information System version 2
DRC: Democratic Republic of the Congo
HSS: Health system strengthening
ITM: Institute of Tropical Medicine
KI: Key informant
KII: Key informant interview
LMICs: Low- and middle-income countries
MHW: Mental health worker
NGOs: Nongovernmental organizations
PCPs: Primary care providers
PHC: Primary health care
PNSM: National Mental Health Program
SDGs: Sustainable development goals
NHIS: National Health Information System
SSA: Sub-Saharan Africa
UHC: Universal Health Coverage
UN: United Nations
WHO: World Health Organization
