# Supplementary material for: Health system facilitators and barriers to the integration of mental health services into primary care in the Democratic Republic of the Congo: a multimethod study
Source: BMC Prim Care. 2024 Jun 13;25:214. doi: 10.1186/s12875-024-02460-y (PMC11170818; doi:10.1186/s12875-024-02460-y)
Supplement: Supplementary file 1 — Additional file 1: Text S1. Interview guide [file 12875_2024_2460_MOESM1_ESM.docx]

**INTERVIEW GUIDE**

| **Introduction** |
| --- |
| Concerns the team of investigators   - Self-presentation (researcher(s)) - Briefly describe the context of the research project - Briefly describe the interview and ask for consent - Explain briefly and ask the following question: Do you give permission for this interview to be recorded? |
| **Warm-up question** |
| - Can you briefly describe your position and role within your organization? |

1. What does "integrating mental health services into a primary care " mean to you?
2. Describe the health system factors (in the DRC) that may hinder the integration of mental health services into primary care in following building blocks:
   1. Leadership and governance
   2. Human resources
   3. Health financing
   4. Medicines and supplies
   5. Service delivery
   6. Health information
   7. Health infrastructure
   8. Population (patient, family, community, culture)
3. Describe the health system factors (in the DRC) likely to facilitate or promote the integration of mental health services into primary care in following building blocks:
   1. Leadership and governance
   2. Human resources
   3. Health financing
   4. Medicines and supplies
   5. Service delivery
   6. Health information
   7. Health infrastructure
   8. Population (patient, family, community)
4. What do you have to talk about our interview?

# Characteristics of the participants

- Participant code:
- Sex of the respondent:
- Province in which the participant currently works:
- Current professional title/status:
- Organization/institution:
- Years of experience:
- Meeting place for the interview

**GUIDE D’ENTRETIEN**

| **Introduction** |
| --- |
| Concerne l’équipe d’enquêteurs   - Auto-présentation (chercheur(s)) - Décrire brièvement le contexte du projet de recherche - Décrire brièvement le déroulement de l'entretien et solliciter le consentement - Expliquer brièvement et poser la question suivante : Autorisez-vous l'enregistrement de cet entretien ? |
| **Question d'échauffement** |
| - Pouvez-vous décrire brièvement votre poste et votre rôle au sein de votre organisation ? |

Que signifie pour vous « l’intégration de la santé mentale dans un service de soins primaires » ?

Décrivez les facteurs de système de santé (en RDC) pouvant s’ériger en obstacles pour freiner l’intégration de la santé mentale dans les services de soins primaires dans les blocs constitutifs suivants :

1. Leadership et gouvernance
2. Ressources humaines
3. Financement de la santé
4. Approvisionnement en médicaments et autres intrants
5. Prestation des services
6. Information sanitaire
7. Infrastructures sanitaires
8. Population (au niveau de patient, de famille, communauté)

Décrivez les facteurs de système de santé (en RDC) susceptibles de faciliter ou favoriser l’intégration de la santé mentale dans les services de soins primaires dans les blocs constitutifs suivants :

1. Leadership et gouvernance
2. Ressources humaines
3. Financement de la santé
4. Approvisionnement en médicaments et autres intrants
5. Prestation des services
6. Information sanitaire
7. Infrastructures sanitaires
8. Population (au niveau patient, de famille, communauté, en rapport avec la culture)

Qu’avez-vous à ajouter à propos de notre entretien ?

**Caractéristiques du (de la) participant(e)**

Code participant :

Sexe du répondant :

Province dans laquelle le participant travaille actuellement :

Titre/Statut professionnel actuel :

Organisation/institution :

Années d’expérience :

Ville de rencontre pour l’entretien
