# Supplementary material for: Health system facilitators and barriers to the integration of mental health services into primary care in the Democratic Republic of the Congo: a multimethod study
Source: BMC Prim Care. 2024 Jun 13;25:214. doi: 10.1186/s12875-024-02460-y (PMC11170818; doi:10.1186/s12875-024-02460-y)
Supplement: Supplementary file 2 — Additional file 2: Text S2. Individual survey questionnaire [file 12875_2024_2460_MOESM2_ESM.docx]

**Individual survey questionnaire**

English version

Code (**#ID**) participant: Name of investigator:

Address codes: Rue , Q/: , C/:

Supervisor name:

Survey date: / /

*This questionnaire is anonymous. Your participation is voluntary.*

**100. Participant profile**

| **N°** | **Socio-professional variables** | **Categories/Answers** | **Code and jump** |
| --- | --- | --- | --- |
| 101 | Type | 1. Male 2. Female | / / |
| 102 | Date of birth (dd/mm/yyyy) | / / | / / years |
| 103 | Highest professional qualification | 1. A2/D6, 2. G3, 3. L2/MD, 4. MPH, 5. PhD | / / |
| 103 | Your main area of experience | 1. Mental health/Psychology/Psychiatry 2. Public health 3. Primary care 4. Administration (Health Sector) 5. Other: ___________________________ | / / |
| 104 | Your business sector | 1. Public 2. Private for-profit 3. Private associations/NGOs | / / |
| 105 | Your professional experience (in years) | / / | / / |

**200 Opinions on integrating mental health into the primary healthcare system**

Based on your knowledge of the Lubumbashi context, tick the box that best describes the following two statements:

1. The integration of mental health into the health center or general hospital is as follows:

| **Not possible** | ☐ | **Probable** | ☐ | **Possible** | ☐ | **Strong possible** | ☐ |
| --- | --- | --- | --- | --- | --- | --- | --- |

1. If mental health is integrated into the health center, screening/diagnosis and management of mental health problems by providers using clinical guidelines will be as follows:

| **Not feasible** | ☐ | **Quite feasible** | ☐ | **Feasible** | ☐ | **Very feasible** | ☐ |
| --- | --- | --- | --- | --- | --- | --- | --- |

**300 Health system facilitators to integrate mental health**

The reasons below are referred to as health system facilitators for the implementation of mental health integration in primary health care (PHC) services. Based on your experience in the field, would you say that the factors listed below can facilitate (promote) the implementation of mental health integration in PHC services? By ticking a box, indicate whether you strongly disagree or strongly agree. **Note**: Please tick only one answer per factor.

| **Strongly disagree** | **Disagree** | **Neither disagree nor agree** | **Agree** | **Strongly agree** |
| --- | --- | --- | --- | --- |
| **1** | **2** | **3** | **4** | **5** |
| ☐ | ☐ | ☐ | ☐ | ☐ |

| **N#** | **Health system facilitators by building blocks** | **Choice of the answer** | | | | |
| --- | --- | --- | --- | --- | --- | --- |
| **3100** | **Governance** | **1** | **2** | **3** | **4** | **5** |
| 3101 | Strong leadership | ☐ | ☐ | ☐ | ☐ | ☐ |
| 3102 | Existence of a frame of reference for senior managers and frontline  staff | ☐ | ☐ | ☐ | ☐ | ☐ |
| 3103 | Strong relationships between service providers and stakeholders (trust,  Respect, informal ties) | ☐ | ☐ | ☐ | ☐ | ☐ |
| 3104 | Communication, coordination/coordinator, effective collaboration | ☐ | ☐ | ☐ | ☐ | ☐ |
| 3105 | Structural and program design facilitators | ☐ | ☐ | ☐ | ☐ | ☐ |
| 3106 | Collective care planning and integration into professional schedules | ☐ | ☐ | ☐ | ☐ | ☐ |
| 3107 | Clear definition of integrated clinical roles | ☐ | ☐ | ☐ | ☐ | ☐ |
| 3108 | Task allocation | ☐ | ☐ | ☐ | ☐ | ☐ |
| 3109 | Political will | ☐ | ☐ | ☐ | ☐ | ☐ |
| 3110 | Commitment of program managers | ☐ | ☐ | ☐ | ☐ | ☐ |
| 3111 | Presence of a mental health service coverage plan | ☐ | ☐ | ☐ | ☐ | ☐ |
| 3112 | Integration guidelines are available or accessible | ☐ | ☐ | ☐ | ☐ | ☐ |
| 3113 | Existence of a Framework for collaboration with other structures | ☐ | ☐ | ☐ | ☐ | ☐ |
| 3114 | Lack of mental health support | ☐ | ☐ | ☐ | ☐ | ☐ |
| 3115 | Good coordination between primary care providers and mental health  health specialists selected for support | ☐ | ☐ | ☐ | ☐ | ☐ |
| **3200** | **Human resources** | **1** | **2** | **3** | **4** | **5** |
| 3201 | Staff training, education, and skills | ☐ | ☐ | ☐ | ☐ | ☐ |
| 3202 | Availability of non-specialist service providers in sufficient numbers | ☐ | ☐ | ☐ | ☐ | ☐ |
| 3203 | Availability of specialized coaching staff | ☐ | ☐ | ☐ | ☐ | ☐ |
| 3204 | Culture, interest, and enthusiasm of staff required/facilitator | ☐ | ☐ | ☐ | ☐ | ☐ |
| 3205 | Collaboration and communication between departments | ☐ | ☐ | ☐ | ☐ | ☐ |
| 3206 | Coordination/navigation (including by a coordinator) | ☐ | ☐ | ☐ | ☐ | ☐ |
| 3207 | Multidisciplinary teams | ☐ | ☐ | ☐ | ☐ | ☐ |
| 3208 | Assigning other human resources to reduce workload | ☐ | ☐ | ☐ | ☐ | ☐ |
| 3209 | Training in Mental Health Integration | ☐ | ☐ | ☐ | ☐ | ☐ |
| 3210 | Limited knowledge of mental disorders 2 | ☐ | ☐ | ☐ | ☐ | ☐ |
| 3211 | Little familiarity with the so-called mental health guidelines | ☐ | ☐ | ☐ | ☐ | ☐ |
| 3212 | Insufficient time to continue referring to the guidelines when integrating  and managing mental health 2 | ☐ | ☐ | ☐ | ☐ | ☐ |
| 3213 | Fear of misdiagnosis | ☐ | ☐ | ☐ | ☐ | ☐ |
| 3214 | Lack of time | ☐ | ☐ | ☐ | ☐ | ☐ |
| 3215 | Availability of a District Mental Health Focal Point for data  management | ☐ | ☐ | ☐ | ☐ | ☐ |
| **3300** | **Medicines and supplies** | **1** | **2** | **3** | **4** | **5** |
| 3301 | Effective drug with minimal side effects | ☐ | ☐ | ☐ | ☐ | ☐ |
| 3302 | Sustainable and uninterrupted supply of medicines | ☐ | ☐ | ☐ | ☐ | ☐ |
| 3303 | Simple treatment regimen | ☐ | ☐ | ☐ | ☐ | ☐ |
| 3304 | Access to equipment and technology (including equipment  maintenance) | ☐ | ☐ | ☐ | ☐ | ☐ |
| 3305 | Inclusion of psychotropic drugs in the essential drug list | ☐ | ☐ | ☐ | ☐ | ☐ |
| 3306 | Existence of a framework for collaboration with trained mental health professionals | ☐ | ☐ | ☐ | ☐ | ☐ |
| 3307 | Existence of a framework dedicated to consultation and observation  and the care of people suffering from mental disorders | ☐ | ☐ | ☐ | ☐ | ☐ |
| **3400** | **Services delivery** | **1** | **2** | **3** | **4** | **5** |
| 3401 | Collaboration between providers within the health department | ☐ | ☐ | ☐ | ☐ | ☐ |
| 3402 | Coordination (of services) and case management | ☐ | ☐ | ☐ | ☐ | ☐ |
| 3403 | Workforce: Staff training and supervision | ☐ | ☐ | ☐ | ☐ | ☐ |
| 3404 | Accessibility: location and setting | ☐ | ☐ | ☐ | ☐ | ☐ |
| 3405 | Techniques/treatment: Rapid reactive support/treatment, tests, and results | ☐ | ☐ | ☐ | ☐ | ☐ |
| 3406 | The fact that general care services are organized | ☐ | ☐ | ☐ | ☐ | ☐ |

| **3500** | **Health financing** | **1** | **2** | **3** | **4** | **5** |
| --- | --- | --- | --- | --- | --- | --- |
| 3501 | Financing, e.g., to pay for installation and maintenance services | ☐ | ☐ | ☐ | ☐ | ☐ |
| 3502 | Insurance coverage and reimbursement to enable customer access  (including for the uninsured) | ☐ | ☐ | ☐ | ☐ | ☐ |
| 3503 | Free care, including medicines | ☐ | ☐ | ☐ | ☐ | ☐ |
| 3504 | Financial incentives for service providers to participate | ☐ | ☐ | ☐ | ☐ | ☐ |
| 3505 | Existence of financial support from development partners | ☐ | ☐ | ☐ | ☐ | ☐ |
| 3506 | Free SMSPS care | ☐ | ☐ | ☐ | ☐ | ☐ |
| **3600** | **Health information** | **1** | **2** | **3** | **4** | **5** |
| 3601 | Existence of mental health indicators in SNIS, defined by the  hierarchy | ☐ | ☐ | ☐ | ☐ | ☐ |
| 3602 | Availability of data collection and reporting tools | ☐ | ☐ | ☐ | ☐ | ☐ |
| 3603 | Availability of appropriate and accessible electronic recording  system | ☐ | ☐ | ☐ | ☐ | ☐ |
| 3604 | Collection/recording of adequate (and high-quality) data | ☐ | ☐ | ☐ | ☐ | ☐ |
| 3605 | Health services connected to the DHIS2 mental health module | ☐ | ☐ | ☐ | ☐ | ☐ |
| **3700** | **Population and context** | **1** | **2** | **3** | **4** | **5** |
| 3701 | Confidence in the effectiveness of formal mental health care | ☐ | ☐ | ☐ | ☐ | ☐ |
| 3702 | Community Participation in Mental Health Activities | ☐ | ☐ | ☐ | ☐ | ☐ |
| 3703 | Family support in identifying mental health problems and seeking  treatment and maintaining attendance | ☐ | ☐ | ☐ | ☐ | ☐ |
| 3704 | Perception that the mentally ill should not receive health care  centers | ☐ | ☐ | ☐ | ☐ | ☐ |
| 3705 | Beliefs about the supernatural causes of mental illness | ☐ | ☐ | ☐ | ☐ | ☐ |
| 3706 | Perception that mental health is everyone’s business | ☐ | ☐ | ☐ | ☐ | ☐ |
| 3707 | Emergence of crises/stressful situations that attract the attention of  decision makers/stakeholders (health and non-health) | ☐ | ☐ | ☐ | ☐ | ☐ |

**400 Health system obstacles to the integration of mental health**

The reasons listed below are referred to as health system barriers to the implementation of mental health integration in primary health care (PHC) services. Based on your experience in the field, would you say that the factors listed below may hinder (prevent) the implementation of the integration of mental health into PHC services? By ticking a box, indicate whether you strongly disagree or strongly agree. **Note**: Please tick only one answer per factor.

| **Strongly disagree** | **No agreement** | **Neither disagree nor agree** | **I agree.** | **I couldn’t agree more.** |
| --- | --- | --- | --- | --- |
| **1** | **2** | **3** | **4** | **5** |
| ☐ | ☐ | ☐ | ☐ | ☐ |

| **N#** | **Obstacles according to blocks** | **Choice of the answer** | | | | |
| --- | --- | --- | --- | --- | --- | --- |
| **4100** | **Governance** | **1** | **2** | **3** | **4** | **5** |
| 4101 | No national mental health policy or plan | ☐ | ☐ | ☐ | ☐ | ☐ |
| 4102 | Lack of a clear integration strategy | ☐ | ☐ | ☐ | ☐ | ☐ |
| 4103 | Lack of priority for mental health within the healthcare system | ☐ | ☐ | ☐ | ☐ | ☐ |
| 4104 | Poor communication between managers and employees regarding mental health issues | ☐ | ☐ | ☐ | ☐ | ☐ |
| 4105 | Complex institutional setup in the design of integrated mental health programs | ☐ | ☐ | ☐ | ☐ | ☐ |
| 4106 | Lack of leadership and commitment from (mental) health managers | ☐ | ☐ | ☐ | ☐ | ☐ |
| 4107 | Resistance to change in Organizational Culture | ☐ | ☐ | ☐ | ☐ | ☐ |
| 4108 | Lack of ongoing training for mental health managers | ☐ | ☐ | ☐ | ☐ | ☐ |
| 4109 | Lack of diversified staff to serve the minority population | ☐ | ☐ | ☐ | ☐ | ☐ |
| 4110 | Lack of mental health supervision | ☐ | ☐ | ☐ | ☐ | ☐ |
| 4111 | Irregularity of district management team meetings with mental health on the agenda | ☐ | ☐ | ☐ | ☐ | ☐ |
| 4112 | Weak involvement of health authorities | ☐ | ☐ | ☐ | ☐ | ☐ |
| 4113 | Vulnerability of the PNSM | ☐ | ☐ | ☐ | ☐ | ☐ |
| 4114 | Weak program coordination | ☐ | ☐ | ☐ | ☐ | ☐ |
| 4115 | Lack of training or standardized guidelines | ☐ | ☐ | ☐ | ☐ | ☐ |
| 4116 | Lack of collaboration with other ministries or government sectors (e.g. police, prisons, education, social welfare and sports) | ☐ | ☐ | ☐ | ☐ | ☐ |
| 4117 | Lack of a mental health manager and/or referent | ☐ | ☐ | ☐ | ☐ | ☐ |
| **4200** | **Human resources** | **1** | **2** | **3** | **4** | **5** |
| 4201 | Staff training, skills, and experience | ☐ | ☐ | ☐ | ☐ | ☐ |

| 4202 | Staff availability | ☐ | ☐ | ☐ | ☐ | ☐ |
| --- | --- | --- | --- | --- | --- | --- |
| 4203 | Lack of staff interest and understanding or acceptance of necessary  behavioral changes | ☐ | ☐ | ☐ | ☐ | ☐ |
| 4204 | Poor collaboration between service providers | ☐ | ☐ | ☐ | ☐ | ☐ |
| 4205 | Lack of specialists to support PHC providers | ☐ | ☐ | ☐ | ☐ | ☐ |
| 4206 | Instability of the current healthcare staff | ☐ | ☐ | ☐ | ☐ | ☐ |
| 4207 | Poor perceived quality of capacity-building activities | ☐ | ☐ | ☐ | ☐ | ☐ |
| 4208 | Fear of misdiagnosis | ☐ | ☐ | ☐ | ☐ | ☐ |
| **4300** | **Medicines and inputs** | **1** | **2** | **3** | **4** | **5** |
| 4301 | Undefined Drug Supply Circuit | ☐ | ☐ | ☐ | ☐ | ☐ |
| 4302 | Lack of essential psychotropic drugs | ☐ | ☐ | ☐ | ☐ | ☐ |
| 4303 | Treatment regimens deemed complex by the providers | ☐ | ☐ | ☐ | ☐ | ☐ |
| 4304 | Lack of appropriate mental health equipment | ☐ | ☐ | ☐ | ☐ | ☐ |
| 4305 | Lack of diagnostic tools, processing, and data collection sheets | ☐ | ☐ | ☐ | ☐ | ☐ |
| 4306 | Absence of patient appointment books | ☐ | ☐ | ☐ | ☐ | ☐ |
| **4400** | **Services** | **1** | **2** | **3** | **4** | **5** |
| 4401 | Referral and link with follow-up treatment | ☐ | ☐ | ☐ | ☐ | ☐ |
| 4402 | Bureaucracy and lack of access to data limit efficiency, monitoring, and evaluation | ☐ | ☐ | ☐ | ☐ | ☐ |
| 4403 | Staff training, skills, and knowledge | ☐ | ☐ | ☐ | ☐ | ☐ |
| 4404 | Resources: Setting up, maintaining, and expanding services, time | ☐ | ☐ | ☐ | ☐ | ☐ |
| 4405 | Lack of knowledge about psychosocial interventions to offer | ☐ | ☐ | ☐ | ☐ | ☐ |
| 4406 | Lack of knowledge about the care process | ☐ | ☐ | ☐ | ☐ | ☐ |
| 4407 | Inability to diagnose and treat mental illness | ☐ | ☐ | ☐ | ☐ | ☐ |
| 4408 | Feeling uncomfortable caring for people with mental health problems | ☐ | ☐ | ☐ | ☐ | ☐ |
| 4409 | Perceived low quality of care SMSPS | ☐ | ☐ | ☐ | ☐ | ☐ |
| 4410 | Burdens associated with traditional service providers | ☐ | ☐ | ☐ | ☐ | ☐ |
| 4411 | Lack of motivation on the part of the MHPSS providers | ☐ | ☐ | ☐ | ☐ | ☐ |
| 4412 | Weak state public services | ☐ | ☐ | ☐ | ☐ | ☐ |
| 4413 | Quality SMSPS care | ☐ | ☐ | ☐ | ☐ | ☐ |
| 4414 | MHPSS complexity and more time | ☐ | ☐ | ☐ | ☐ | ☐ |
| **4500** | **Financing** | **1** | **2** | **3** | **4** | **5** |
| 4501 | Set-up, maintenance, and expansion costs (e.g. because more people have been identified) | ☐ | ☐ | ☐ | ☐ | ☐ |

| 4502 | Insurance coverage | ☐ | ☐ | ☐ | ☐ | ☐ |
| --- | --- | --- | --- | --- | --- | --- |
| 4503 | Low/lack of a separate state budget for mental health | ☐ | ☐ | ☐ | ☐ | ☐ |
| 4504 | Direct Payment for Mental Health Care by Patients/Families | ☐ | ☐ | ☐ | ☐ | ☐ |
| 4505 | High cost of hiring and supporting specialists | ☐ | ☐ | ☐ | ☐ | ☐ |
| 4506 | Inexistence or insufficient mental health insurance coverage | ☐ | ☐ | ☐ | ☐ | ☐ |
| 4507 | Financing SMSPS | ☐ | ☐ | ☐ | ☐ | ☐ |
| 4508 | Unprofitable MHPSS service | ☐ | ☐ | ☐ | ☐ | ☐ |
| 4509 | Cost of recruiting new specialized service providers | ☐ | ☐ | ☐ | ☐ | ☐ |
| **4600** | **Information** | **1** | **2** | **3** | **4** | **5** |
| 4601 | Insufficient/mediocre data collection | ☐ | ☐ | ☐ | ☐ | ☐ |
| 4602 | Data access difficulties due to Different Supplier Systems | ☐ | ☐ | ☐ | ☐ | ☐ |
| 4603 | Lack of reporting templates | ☐ | ☐ | ☐ | ☐ | ☐ |
| 4604 | Mental health module not connected to DHIS2 | ☐ | ☐ | ☐ | ☐ | ☐ |
| 4605 | Low quality/quantity of data (information) reported | ☐ | ☐ | ☐ | ☐ | ☐ |
| 4606 | No monitoring system | ☐ | ☐ | ☐ | ☐ | ☐ |
| **4700** | **Population** | **1** | **2** | **3** | **4** | **5** |
| 4701 | Belief in the efficacy of traditional healers | ☐ | ☐ | ☐ | ☐ | ☐ |
| 4702 | Belief in the efficacy of spiritual healers | ☐ | ☐ | ☐ | ☐ | ☐ |
| 4703 | Belief in the supernatural causes of mental illness | ☐ | ☐ | ☐ | ☐ | ☐ |
| 4704 | Political and individual rights to mental health are not guaranteed | ☐ | ☐ | ☐ | ☐ | ☐ |
| 4705 | The fact that the public is not protected against mental illness | ☐ | ☐ | ☐ | ☐ | ☐ |
| 4706 | Stigmatization of mental health | ☐ | ☐ | ☐ | ☐ | ☐ |
| 4707 | No community ownership | ☐ | ☐ | ☐ | ☐ | ☐ |
| 4708 | Cultural considerations | ☐ | ☐ | ☐ | ☐ | ☐ |
| 4709 | High levels of stigmatization | ☐ | ☐ | ☐ | ☐ | ☐ |
| 4710 | Poverty among the sick | ☐ | ☐ | ☐ | ☐ | ☐ |
| 4711 | Perception that formal mental health care will be expensive | ☐ | ☐ | ☐ | ☐ | ☐ |
| 4712 | Perception that Western medical treatment for mental illness is harmful and unnecessary | ☐ | ☐ | ☐ | ☐ | ☐ |
| 4713 | Confidentiality concerns | ☐ | ☐ | ☐ | ☐ | ☐ |
| 4714 | Lack of involvement of traditional and spiritual healers | ☐ | ☐ | ☐ | ☐ | ☐ |

Investigator’s signature

**Questionnaire d’enquête individuelle**

Version française

| Code (**#ID**) participant : ______________________ | Nom enquêteur : ___________ |
| --- | --- |
| Codes Adresse : Rue_____, Q/ : ___________, C/: ___________ | Nom superviseur : __________ |
|  | Date enquête : ____/____/___ |

*Ce questionnaire est anonyme. Votre participation volontaire souhaitée.*

**100. Profil des participants**

| **N°** | **Variables socioprofessionnelles** | **Catégories/Réponses** | **Code et saut** |
| --- | --- | --- | --- |
| 101 | Genre | 1. Masculin 2. Féminin | /_____/ |
| 102 | Date de naissance (jj/mm/aaaa) | _____ /______/______ | /_____/ ans |
| 103 | Diplôme professionnel le plus élevé | 1. A2/D6, 2. G3, 3. L2/MD, 4. MPH, 5. PhD | /_____/ |
| 103 | Votre principal domaine d’expérience | 1. Santé mentale/Psychologie/Psychiatrie  2. Santé publique 3. Soins de santé primaires  4. Administration (Secteur Santé) | /_____/ |
| 104 | Votre secteur d’activités | 1. Public  2. Privé lucratif  3. Privé associatif/ONGs | /_____/ |
| 105 | Votre expérience professionnelle (en années) | /_____/ | /_____/ |

**200. Opinions sur l’intégration de la santé mentale dans le système des soins de santé primaires**

D’après votre connaissance du contexte de Lubumbashi, cochez la case qui correspond le mieux aux deux énoncés suivants :

1. L’intégration de la santé mentale au centre de santé ou à l’hôpital général est :

| **Pas possible** |  | **Probable** |  | **Possible** |  | **Fort possible** |  |
| --- | --- | --- | --- | --- | --- | --- | --- |

2. En cas d’intégration de la santé mentale au centre de santé, le dépistage/diagnostic et la prise en charge des problèmes de santé mentale par les prestataires à l’aide des lignes directrices cliniques sera :

| **Pas faisable** |  | **Assez faisable** |  | **Faisable** |  | **Très faisable** |  |
| --- | --- | --- | --- | --- | --- | --- | --- |

**300. Facilitateurs du système de santé à la mise en œuvre de l’intégration de santé mentale**

Les motifs ci-dessous sont appelés facilitateurs du système de santé à la mise en œuvre de l’intégration de la santé mentale dans des services de soins de santé primaires (SSP). D’après votre expérience du milieu, direz-vous que les facteurs indiqués ci-dessous peuvent faciliter (favoriser) la mise en œuvre de l’intégration de la santé mentale dans les services de SSP ? Indiquez en cochant une case si vous n’êtes pas du tout d’accord ou vous êtes tout à fait d’accord. **Note** : Veuillez cocher une seule réponse par facteur.

| **Pas du tout d’accord** | **Pas d’accord** | **Ni en désaccord ni d'accord** | **D’accord** | **Tout à fait d’accord** |
| --- | --- | --- | --- | --- |
| **1** | **2** | **3** | **4** | **5** |
|  |  |  |  |  |

| **N#** | **Facilitateurs en fonction de blocks constitutifs du système de santé** | **Choix de réponse** | | | | |
| --- | --- | --- | --- | --- | --- | --- |
| **3100** | **Gouvernance** | **1** | **2** | **3** | **4** | **5** |
| 3101 | Leadership fort |  |  |  |  |  |
| 3102 | Existence d’un cadre de référence pour des cadres supérieurs et le personnel de première ligne |  |  |  |  |  |
| 3103 | Des relations solides entre les prestataires et les parties prenantes (confiance, respect, liens informels) |  |  |  |  |  |
| 3104 | Communication, Coordination/coordinateur, Collaboration efficace |  |  |  |  |  |
| 3105 | Facilitateurs de conception structurelle et de programme |  |  |  |  |  |
| 3106 | Planification collective des soins et leur intégration dans les horaires des professionnels |  |  |  |  |  |
| 3107 | Définition claire des rôles cliniques intégrés |  |  |  |  |  |
| 3108 | Répartition des tâches |  |  |  |  |  |
| 3109 | La volonté politique |  |  |  |  |  |
| 3110 | L’engagement des gestionnaires du programme |  |  |  |  |  |
| 3111 | Présence d’un plan de couverture en services de santé mentale |  |  |  |  |  |
| 3112 | Les lignes directrices sur l’intégration disponibles ou accessibles |  |  |  |  |  |
| 3113 | Existence d’un cadre de collaboration avec d’autres structures |  |  |  |  |  |
| 3114 | Le manque de soutien (du Programme national de santé mentale, de la DPS, du BCZS, et de la communauté) en santé mentale |  |  |  |  |  |
| 3115 | Bonne coordination entre les prestataires de soins primaires et les spécialistes de santé mentale choisis pour l’accompagnement |  |  |  |  |  |
| **3200** | **Ressources humaines** | **1** | **2** | **3** | **4** | **5** |
| 3201 | Formation, éducation, compétences du personnel |  |  |  |  |  |
| 3202 | Disponibilité des prestataires non-spécialistes en nombre suffisant |  |  |  |  |  |
| 3203 | Disponibilité du personnel spécialisé chargé du coaching |  |  |  |  |  |
| 3204 | Culture, intérêt, enthousiasme du personnel nécessaire/facilitateur |  |  |  |  |  |
| 3205 | Collaboration/communication entre les services |  |  |  |  |  |
| 3206 | Coordination/navigation (y compris par un coordinateur) |  |  |  |  |  |
| 3207 | Des équipes multidisciplinaires |  |  |  |  |  |
| 3208 | Le fait d’affecter d’autres ressources humaines pour réduire la charge de travail |  |  |  |  |  |
| 3209 | La formation à l’intégration de la santé mentale |  |  |  |  |  |
| 3210 | Les connaissances limitées sur les troubles mentaux 2 |  |  |  |  |  |
| 3211 | Peu de familiarité avec les lignes directrices dites sur la santé mentale |  |  |  |  |  |
| 3212 | Temps insuffisant pour continuer à se référer aux lignes directrices lors d’intégration et de la prise en charge de la santé mentale 2 |  |  |  |  |  |
| 3213 | La peur de faire un mauvais diagnostic |  |  |  |  |  |
| 3214 | Le manque de temps |  |  |  |  |  |
| 3215 | Disponibilité d’un point focal santé mentale du district pour la gestion des données |  |  |  |  |  |
| **3300** | **Médicaments et intrants** | **1** | **2** | **3** | **4** | **5** |
| 3301 | Médicament efficace, minimisant les effets indésirables |  |  |  |  |  |
| 3302 | Approvisionnement durable et ininterrompu en médicaments |  |  |  |  |  |
| 3303 | Régime de traitement simple |  |  |  |  |  |
| 3304 | Accès à l'équipement et à la technologie (y compris l'entretien de l'équipement) |  |  |  |  |  |
| 3305 | Inclusion de médicaments psychotropes dans la liste des médicaments essentiels |  |  |  |  |  |
| 3306 | Existence d’un cadre de collaboration avec un personnel formé en santé mentale |  |  |  |  |  |
| 3307 | Existence d’un cadre de travail dédié à la consultation, l’observation et la prise en charge des personnes souffrant de troubles mentaux |  |  |  |  |  |
| **3400** | **Prestations de services** | **1** | **2** | **3** | **4** | **5** |
| 3401 | Collaboration entre prestataires au sein du service de santé |  |  |  |  |  |
| 3402 | Coordination (des services) et gestion de cas |  |  |  |  |  |
| 3403 | Main-d'œuvre - formation et supervision du personnel |  |  |  |  |  |
| 3404 | Accessibilité - emplacement, cadre |  |  |  |  |  |
| 3405 | Techniques/traitement - soutien/traitement réactif rapide, tests, résultats |  |  |  |  |  |
| 3406 | Le fait que les services généraux de soins soient organisés |  |  |  |  |  |
| **3500** | **Financement** | **1** | **2** | **3** | **4** | **5** |
| 3501 | Financement, par ex. pour payer les services de mise en place et de maintien |  |  |  |  |  |
| 3502 | Couverture d'assurance et remboursement pour permettre l'accès des clients (y compris pour les non-assurés) |  |  |  |  |  |
| 3503 | Soins gratuits, y compris les médicaments |  |  |  |  |  |
| 3504 | Incitations financières pour les prestataires à participer |  |  |  |  |  |
| 3505 | Existence d’un appui financier des partenaires au développement |  |  |  |  |  |
| 3506 | Gratuité des soins de SMSPS |  |  |  |  |  |
| **3600** | **Informations** | **1** | **2** | **3** | **4** | **5** |
| 3601 | Existence des indicateurs de santé mentale dans le SNIS, définis par la hiérarchie |  |  |  |  |  |
| 3602 | Disponibilité des outils de collecte et rapportage des données |  |  |  |  |  |
| 3603 | Disponibilité d’un système d’enregistrement électronique appropriés et accessibles |  |  |  |  |  |
| 3604 | Collecte/enregistrement de données adéquates (et de qualité adéquate) |  |  |  |  |  |
| 3605 | Services de santé connectés au module santé mentale du DHIS2 |  |  |  |  |  |
| **3700** | **Population et contexte** | **1** | **2** | **3** | **4** | **5** |
| 3701 | Confiance en l’efficacité des soins formels de santé mentale |  |  |  |  |  |
| 3702 | Participation communautaire aux activités de santé mentale |  |  |  |  |  |
| 3703 | Soutien familial pour la détection des problèmes de santé mentale, la recherche du traitement et l’assiduité |  |  |  |  |  |
| 3704 | Perception que les malades mentaux ne devraient pas recevoir de soins en centres de santé |  |  |  |  |  |
| 3705 | Les croyances sur les causes surnaturelles de la maladie mentale |  |  |  |  |  |
| 3706 | Perception selon laquelle la santé mentale est l’apanage de tous |  |  |  |  |  |
| 3707 | L’émergence des crises/situations stressantes qui attirent l’attention des décideurs/acteurs (sanitaires et non sanitaires) |  |  |  |  |  |

**400. Obstacles du système de santé à la mise en œuvre de l’intégration de santé mentale**

Les motifs ci-dessous sont appelés obstacles du système de santé à la mise en œuvre de l’intégration de la santé mentale dans des services de soins de santé primaires (SSP). D’après votre expérience du milieu, direz-vous que les facteurs indiqués ci-dessous peuvent freiner (empêcher) la mise en œuvre de l’intégration de la santé mentale dans les services de SSP ? Indiquez en cochant une case si vous n’êtes pas du tout d’accord ou vous êtes tout à fait d’accord. **Note** : Veuillez cocher une seule réponse par facteur.

| **Pas du tout d’accord** | **Pas d’accord** | **Ni en désaccord ni d'accord** | **D’accord** | **Tout à fait d’accord** |
| --- | --- | --- | --- | --- |
| **1** | **2** | **3** | **4** | **5** |
|  |  |  |  |  |

| **N#** | **Obstacles en fonction de blocks** | **Choix de réponse** | | | | |
| --- | --- | --- | --- | --- | --- | --- |
| **4100** | **Gouvernance** | **1** | **2** | **3** | **4** | **5** |
| 4101 | Absence de politique ou de plan national de santé mentale |  |  |  |  |  |
| 4102 | Absence d’une stratégie d’intégration claire |  |  |  |  |  |
| 4103 | Absence de priorité de la santé mentale au sein du système de santé |  |  |  |  |  |
| 4104 | Communication déficiente entre gestionnaires et collaborateurs sur la question de santé mentale |  |  |  |  |  |
| 4105 | Montage institutionnel complexe dans la conception de programme d’intégration de santé mentale |  |  |  |  |  |
| 4106 | Manque de leadership et d'engagement des gestionnaires de la santé (mentale) |  |  |  |  |  |
| 4107 | Résistance au changement de culture organisationnelle |  |  |  |  |  |
| 4108 | Manque de formation continue des gestionnaires en santé mentale |  |  |  |  |  |
| 4109 | Manque de personnel diversifié pour desservir la minorité |  |  |  |  |  |
| 4110 | Manque des supervisions santé mentale |  |  |  |  |  |
| 4111 | Irrégularité des réunions des équipes cadres des districts avec ordre de jour intégrant la santé mentale |  |  |  |  |  |
| 4112 | Faible implication des autorités sanitaires |  |  |  |  |  |
| 4113 | Vulnérabilité du PNSM |  |  |  |  |  |
| 4114 | Faible coordination du programme |  |  |  |  |  |
| 4115 | Absence de formation ou de lignes directrices normalisées |  |  |  |  |  |
| 4116 | Absence de collaborations avec d'autres ministères ou secteurs gouvernementaux (par exemple, la police, la prison, l'éducation, la protection sociale et les sports) sur la thématique d’intégration |  |  |  |  |  |
| 4117 | Absence d'un responsable et/ou d'un référent en santé mentale |  |  |  |  |  |
| **4200** | **Ressources humaines** | **1** | **2** | **3** | **4** | **5** |
| 4201 | Formation, compétences et expérience du personnel |  |  |  |  |  |
| 4202 | Disponibilité du personnel |  |  |  |  |  |
| 4203 | Manque d'intérêt du personnel, de compréhension ou d'acceptation des changements de comportement nécessaires |  |  |  |  |  |
| 4204 | Mauvaise collaboration entre les prestataires |  |  |  |  |  |
| 4205 | Manque de spécialistes pour accompagner les prestataires de SSP |  |  |  |  |  |
| 4206 | Instabilité du personnel de santé en poste |  |  |  |  |  |
| 4207 | Mauvaise qualité perçue des activités de renforcement des capacités |  |  |  |  |  |
| 4208 | La peur de faire un mauvais diagnostic |  |  |  |  |  |
| **4300** | **Médicaments et intrants** | **1** | **2** | **3** | **4** | **5** |
| 4301 | Circuit d’approvisionnement en médicaments non défini |  |  |  |  |  |
| 4302 | Manque de médicaments psychotropes essentiels |  |  |  |  |  |
| 4303 | Schémas thérapeutiques jugés complexes par les prestataires |  |  |  |  |  |
| 4304 | Manque d'équipement de santé mentale approprié |  |  |  |  |  |
| 4305 | Absence des outils de diagnostic, traitement et fiches de collecte des données |  |  |  |  |  |
| 4306 | Absence des carnets de rendez-vous patients |  |  |  |  |  |
| **4400** | **Prestations de services** | **1** | **2** | **3** | **4** | **5** |
| 4401 | Orientation et lien avec le traitement de suivi |  |  |  |  |  |
| 4402 | La bureaucratie, le manque d'accès aux données limitent l'efficacité, le suivi et l'évaluation |  |  |  |  |  |
| 4403 | Formation, compétences et connaissances du personnel |  |  |  |  |  |
| 4404 | Ressources – mise en place, maintien et expansion des services, temps |  |  |  |  |  |
| 4405 | Manque de connaissances sur les interventions psychosociales à offrir |  |  |  |  |  |
| 4406 | Manque de connaissances sur la démarche de prise en charge |  |  |  |  |  |
| 4407 | Incapacité à diagnostiquer et à traiter une maladie mentale |  |  |  |  |  |
| 4408 | Le fait de se sentir mal à l’aise de s’occuper de personnes souffrant de troubles mentaux |  |  |  |  |  |
| 4409 | Faible qualité de soins SMSPS perçue |  |  |  |  |  |
| 4410 | Pesanteurs liées aux prestataires traditionnels |  |  |  |  |  |
| 4411 | Prestataires pas très motivés pour la SMSPS |  |  |  |  |  |
| 4412 | Faiblesse des services publics de l’Etat |  |  |  |  |  |
| 4413 | Offre de soins SMSPS de qualité |  |  |  |  |  |
| 4414 | Complexité des SMSPS, plus de temps |  |  |  |  |  |
| **4500** | **Financement** | **1** | **2** | **3** | **4** | **5** |
| 4501 | Coûts de mise en place, de maintien et d'expansion (par exemple, parce que plus de personnes ont été identifiées) |  |  |  |  |  |
| 4502 | Couverture d'assurance |  |  |  |  |  |
| 4503 | Faible/manque de budget de l’Etat séparé pour la santé mentale |  |  |  |  |  |
| 4504 | Paiement direct des soins de santé mentale par les patients/familles |  |  |  |  |  |
| 4505 | Coût élevé alloué à l’engagement et au soutien des spécialistes |  |  |  |  |  |
| 4506 | Inexistence/insuffisante de couverture d’assurance maladie mentale |  |  |  |  |  |
| 4507 | Financement SMSPS |  |  |  |  |  |
| 4508 | Service de SMSPS peu rentable |  |  |  |  |  |
| 4509 | Coût de recrutement de nouveaux prestataires spécialisés |  |  |  |  |  |
| **4600** | **Information** | **1** | **2** | **3** | **4** | **5** |
| 4601 | Collecte de données insuffisante/médiocre |  |  |  |  |  |
| 4602 | Difficultés d'accès aux données dues aux différents systèmes de fournisseurs |  |  |  |  |  |
| 4603 | Absence de canevas de rapportage |  |  |  |  |  |
| 4604 | Manque de connexion du module santé mentale au DHIS2 |  |  |  |  |  |
| 4605 | Faible qualité/quantité de données (informations) rapportées |  |  |  |  |  |
| 4606 | Absence du système de monitoring |  |  |  |  |  |
| **4700** | **Population** | **1** | **2** | **3** | **4** | **5** |
| 4701 | Croyance en l’efficacité des guérisseurs traditionnels |  |  |  |  |  |
| 4702 | Croyance en l’efficacité des guérisseurs spirituels |  |  |  |  |  |
| 4703 | Croyance en des causes surnaturelles de maladie mentale |  |  |  |  |  |
| 4704 | Le fait que les droits politiques et individuels en matière de santé mentale ne sont pas garantis |  |  |  |  |  |
| 4705 | Le fait que le public n’est pas protégé contre les malades mentaux |  |  |  |  |  |
| 4706 | Stigmatisation de la santé mentale |  |  |  |  |  |
| 4707 | Non appropriation de la communauté |  |  |  |  |  |
| 4708 | Considérations culturelles |  |  |  |  |  |
| 4709 | Niveaux élevés de stigmatisation |  |  |  |  |  |
| 4710 | Pauvreté des malades |  |  |  |  |  |
| 4711 | Perception que les soins formels de santé mentale couteront cher |  |  |  |  |  |
| 4712 | Perception que le traitement d’une maladie mentale dans la médecine occidentale est nocif et inutile |  |  |  |  |  |
| 4713 | Préoccupations concernant la confidentialité |  |  |  |  |  |
| 4714 | Absence d'engagement des guérisseurs traditionnels et spirituels |  |  |  |  |  |

Signature de l’enquêteur
